# Global REnal Involvement of CORonavirus Disease 2019 (RECORD): A Systematic Review and Meta-Analysis of Incidence, Risk Factors, and Clinical Outcomes

**DOI:** 10.3389/fmed.2021.678200

**Published:** 2021-05-25

**Authors:** Kam Wa Chan, Kam Yan Yu, Pak Wing Lee, Kar Neng Lai, Sydney Chi-Wai Tang

**Affiliations:** ^1^Department of Medicine, Queen Mary Hospital, The University of Hong Kong, Hong Kong, China; ^2^Faculty of Epidemiology and Population Health, London School of Hygiene & Tropical Medicine, London, United Kingdom

**Keywords:** COVID-19, acute kidney injury, renal medicine, meta-analysis, risk factor, meta-regression, systematic review, internal medicine

## Abstract

**Introduction:** The quantitative effect of underlying non-communicable diseases on acute kidney injury (AKI) incidence and the factors affecting the odds of death among coronavirus disease 2019 (COVID-19) AKI patients were unclear at population level. This study aimed to assess the association between AKI, mortality, underlying non-communicable diseases, and clinical risk factors.

**Methods:** A systematic search of six databases was performed from January 1, 2020, until October 5, 2020. Peer-reviewed observational studies containing quantitative data on risk factors and incidence of renal manifestations of COVID-19 were included. Location, institution, and time period were matched to avoid duplicated data source. Incidence, prevalence, and odds ratio of outcomes were extracted and pooled by random-effects meta-analysis. History of renal replacement therapy (RRT) and age group were stratified for analysis. Univariable meta-regression models were built using AKI incidence as dependent variable, with underlying comorbidities and clinical presentations at admission as independent variables.

**Results:** Global incidence rates of AKI and RRT in COVID-19 patients were 20.40% [95% confidence interval (CI) = 12.07–28.74] and 2.97% (95% CI = 1.91–4.04), respectively, among patients without RRT history. Patients who developed AKI during hospitalization were associated with 8 times (pooled OR = 9.03, 95% CI = 5.45–14.94) and 16.6 times (pooled OR = 17.58, 95% CI = 10.51–29.38) increased odds of death or being critical. At population level, each percentage increase in the underlying prevalence of diabetes, hypertension, chronic kidney disease, and tumor history was associated with 0.82% (95% CI = 0.40–1.24), 0.48% (95% CI = 0.18–0.78), 0.99% (95% CI = 0.18–1.79), and 2.85% (95% CI = 0.93–4.76) increased incidence of AKI across different settings, respectively. Although patients who had a kidney transplant presented with a higher incidence of AKI and RRT, their odds of mortality was lower. A positive trend of increased odds of death among AKI patients against the interval between symptom onset and hospital admission was observed.

**Conclusion:** Underlying prevalence of non-communicable diseases partly explained the heterogeneity in the AKI incidence at population level. Delay in admission after symptom onset could be associated with higher mortality among patients who developed AKI and warrants further research.

## Introduction

As of April 6, 2021, coronavirus disease 2019 (COVID-19) has infected 132 million people, leading to 2.9 million deaths, and the challenge of the long COVID-19 symptoms is emerging (1). As the incidence of COVID-19 is expected to substantially reduce after vaccination becomes accessible, the admission policy in different jurisdiction could be more flexible to screen or hospitalize patients to prevent serious outcomes, for instance, acute kidney injury (AKI) and the associated long-term sequelae (2).

The global renal involvement, including the related risk of death and the association with underlying comorbidities, among COVID-19 patients is relatively less well-characterized (2–4). Previous studies from the United States reported the incidence of AKI to reach 56.9% (5), whereas studies from China reported the AKI incidence to be <10% (6, 7). The discrepancy in the reported incidence of AKI and other renal manifestations could be due to the differences in screening and admission policy in different jurisdictions, which led to varying demographics among cohorts (8). The variation in demographics between countries and cohorts provided an opportunity to assess the correlation between the prevalence of underlying non-communicable diseases and the incidence of AKI globally, which remains unclear (9, 10).

In-hospital AKI is associated with lengthened hospital stay (11), increased utilization of critical care beds (12), healthcare expenditure (11, 13), mortality (12, 14), risk of subsequent chronic kidney disease, end-stage kidney disease (15), *de novo* diabetes (16), and *de novo* hypertension (17) with limited effective regimens (18). The factors associated with faster and better recovery of AKI in COVID-19 remain unclear. The current strategy in the prevention of AKI mainly lies on early identification and correction of abnormal fluid balance. Therefore, whether earlier admission with prompt volume correction could reduce AKI-associated mortality among COVID-19 patients requires urgent assessment.

Cytokine storm and elevated d-dimer levels have been widely reported and suspected to correlate with poorer clinical outcomes (8, 19–21). Previous renal pathology studies showed that COVID-19 patients with clinical signs of kidney injuries had diffuse proximal tubule injury, loss of brush border, non-isometric vacuolar degeneration, and gross necrosis with possible association with systemic hypoxia, abnormal coagulation, and rhabdomyolysis (22). Cytokine storm and microangiopathy with hypercoagulability are hypothesized to be the key underlying mechanisms of AKI in COVID-19 on top of direct viral infection, associated hemodynamic changes, and secondary damage related to multiple organ dysfunction (3, 23–25).

We aimed to assess the renal involvement in COVID-19 patients worldwide including the associated risk of death. We also assessed the association of AKI with underlying non-communicable diseases and identified factors associated with increased odds of death among AKI patients.

## Methods

### Study Design

This was a systematic review and meta-analysis with meta-regression. The review protocol was prospectively registered on PROSPERO (CRD42020184621).

### Literature Search

We sought to assess the odds ratio (OR) of different risk factors associated with AKI, renal replacement therapy (RRT) and mortality, and the incidence of AKI and RRT in any settings. The search strategy (Supplementary 1) was formulated to include all clinical studies containing original data on risk factors and incidence of renal manifestations published since the outbreak of COVID-19, until October 5, 2020. We searched through six databases: Cochrane, MEDLINE, EMBASE, PubMed, CNKI (China National Knowledge Infrastructure), and Wanfang Data. Reference lists and studies included in systematic reviews were also searched. Only studies with COVID-19 patients confirmed by reverse transcription–polymerase chain reaction were included. A team with clinical epidemiologist (K.W.C.) led the search and data processing. Endnote X9 was used to aid the review process.

### Outcome Measurement

Primary outcomes included incidence of in-hospital AKI and RRT. Secondary outcomes included weighed mean difference (WMD), standardized mean difference (SMD), and ORs of different risk factors associated with the primary outcomes and mortality. Patients with different history of RRT and age group were stratified.

### Screening

Screening started with title and abstract followed by full text before data extraction. All articles were duly screened (P.W.L., K.Y.Y., K.W.C.), assessed (P.W.L., K.W.C.), and extracted (K.Y.Y., K.W.C.) independently with a standardized form. All disagreement was resolved by discussion. There was no language restriction. Observational studies (including case series, cohort study, and case–control study) containing quantitative data on risk factors and incidence of renal manifestations published since the outbreak of COVID-19 were included. All single case reports, study protocols, clinical trials, qualitative studies, and studies with no quantitative COVID-related data were excluded. Non-English and non-Chinese articles were translated to English before assessment.

When an original study with the same data source was reported by multiple studies, only the one with the most complete data was included. To avoid duplicated data source from different publications, we matched the location, institution, and time period and included only the data source of each outcome based on the following priority: (1) with clear outcome definition, (2) consecutive sampling, and (3) sample size, from potentially duplicated patient source.

### Quality Assessment

Quality of included studies was assessed by the National Institutes of Health (NIH) Study Quality Assessment Tools according to the study design (26), to evaluate the risk of bias and to assess the overall validity of reported results. Studies with overall poor quality (scored 3 or lower from a 9-point scale) were defined as having a high risk of bias. Studies with overall high quality (scored 7 or greater from a 9-point scale) were defined as having a low risk of bias.

### Data Extraction and Meta-Analysis

In the meta-analysis of incidence of renal manifestations, we removed studies with duplicated source and included only studies sampled from the general population and indicated consecutive or random sampling to ensure representative sampling. The incidence and prevalence of outcomes were expressed in pooled percentages. ORs were expressed as pooled ORs with 95% confidence intervals (CIs) using random-effects model and presented with forest plots. As renal manifestation is expected to vary considerably across settings because of different local screening and management protocols, we used a random-effects model in the main analysis supplemented by the fixed-effects model as the sensitivity analysis. Patients with RRT history were excluded from the pooled analysis.

Pooled quantitative variables were presented with WMD, and SMD with magnitude regarded as small, medium, and high corresponded to SMD = 0.2, 0.5, and 0.8, respectively (27). All medians were transformed to means for meta-analysis (28). *I*^2^ statistic was calculated to measure the proportion of total variation in study estimates attributed to heterogeneity. *I*^2^ values of 0 to 40%, 30 to 60%, 50 to 90%, and 75 to 100% indicated that heterogeneity was (1) unlikely to be important, (2) moderate, (3) substantial, and (4) considerable, respectively (29). Sensitivity analyses were performed with fixed-effects model. Subgroup analyses were performed by stratifying city of study, kidney transplant history, and pediatric patients. Only studies with clear definition of AKI [e.g., Kidney Disease Improving Global Outcomes (KDIGO)] were included in the AKI-related analysis.

### Meta-Regression

Univariable meta-regression models were built using the incidence of AKI as dependent variable, with underlying comorbidities and clinical presentations at admission as independent variables to assess the association between the prevalence of non-communicable diseases in various regions, clinical presentations at admission, and the incidence of AKI during COVID, from a public health perspective at the regional level. The *p*-value was computed by Monte Carlo test with 3,000 permutations. All analyses were conducted using STATA 15.1.

## Results

**Figure 1:**
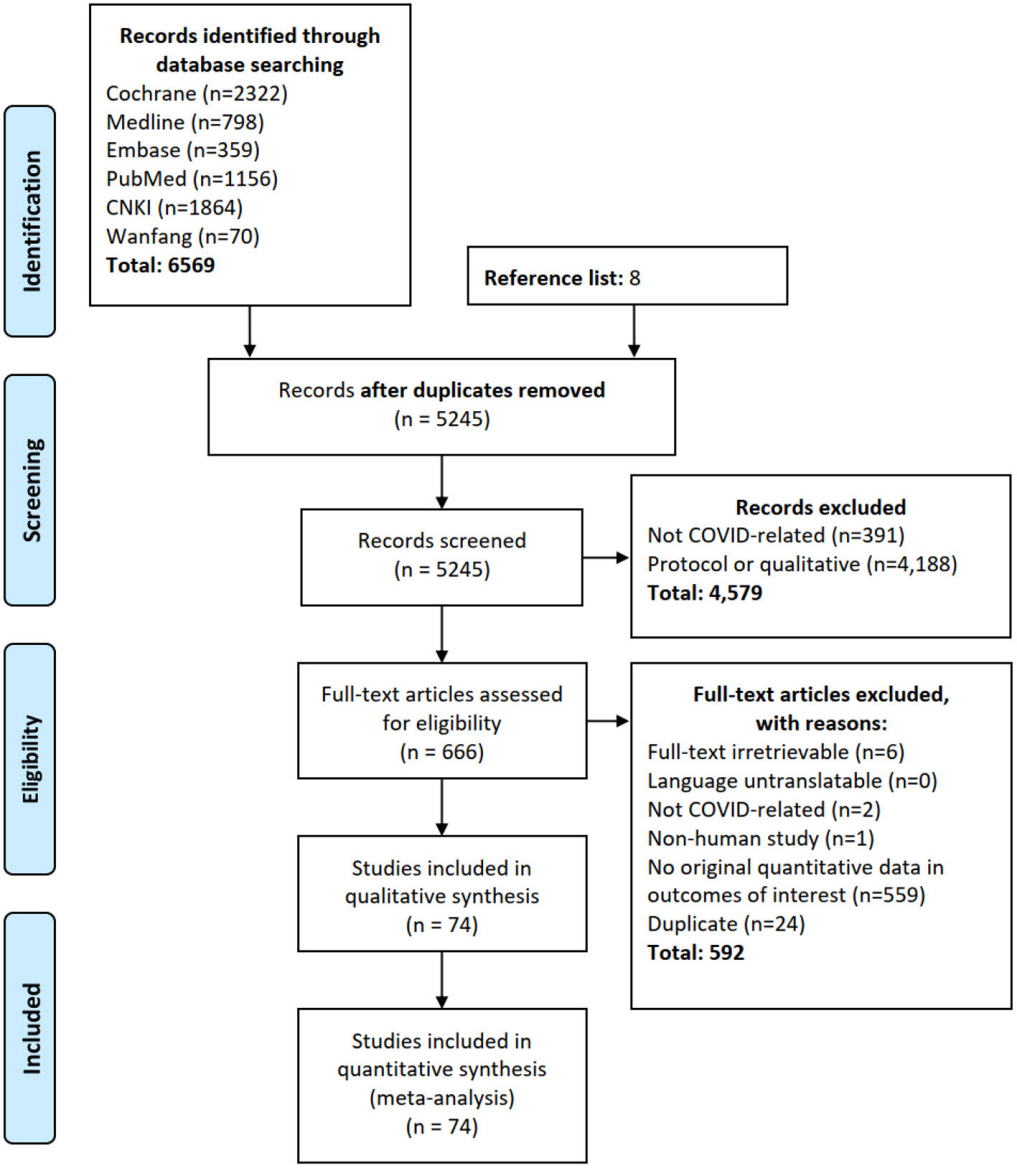
Flow diagram of literature search. Our search identified 5,245 deduplicated studies from six databases (*n* = 6,569) and reference list search (*n* = 8). Seventy-four studies from more than 60 provinces/states of 17 countries contained quantitative data on the renal manifestations and were included.

The flow of systematic review and meta-analysis is summarized in Figure 1. Our search identified 5,245 deduplicated studies from six databases (*n* = 6,569) and reference list search (*n* = 8). Seventy-four studies from more than 60 provinces/states of Austria, Brazil, Canada, China, Denmark, France, Germany, India, Iran, Italy, Japan, Korea, Malaysia, Spain, Turkey, United Kingdom, and United States contained quantitative data on the renal manifestations and were included. Excluded studies are listed in Supplementary 2, and the characteristics of included studies are summarized in Supplementary 3.

Five prospective cohorts, 67 retrospective cohorts/registries, and 2 case–control studies were included. Forty-three studies included COVID-19 patients from the general population. Forty studies indicated consecutive or random sampling. The median sample size of the included studies was 101 [interquartile range (IQR) = 36–379]. The median follow-up period was 4 weeks (IQR = 2–4). Overall, the study quality was satisfactory with 49 and 25 studies attaining 7 to 9 and 4 to 6 out of the 9-point NIH assessment tool (Supplementary 4) (26). The majority of studies reported AKI according to the KDIGO 2012 guideline (30).

### Global Burden of Renal Manifestations From COVID-19

**Figure 2:**
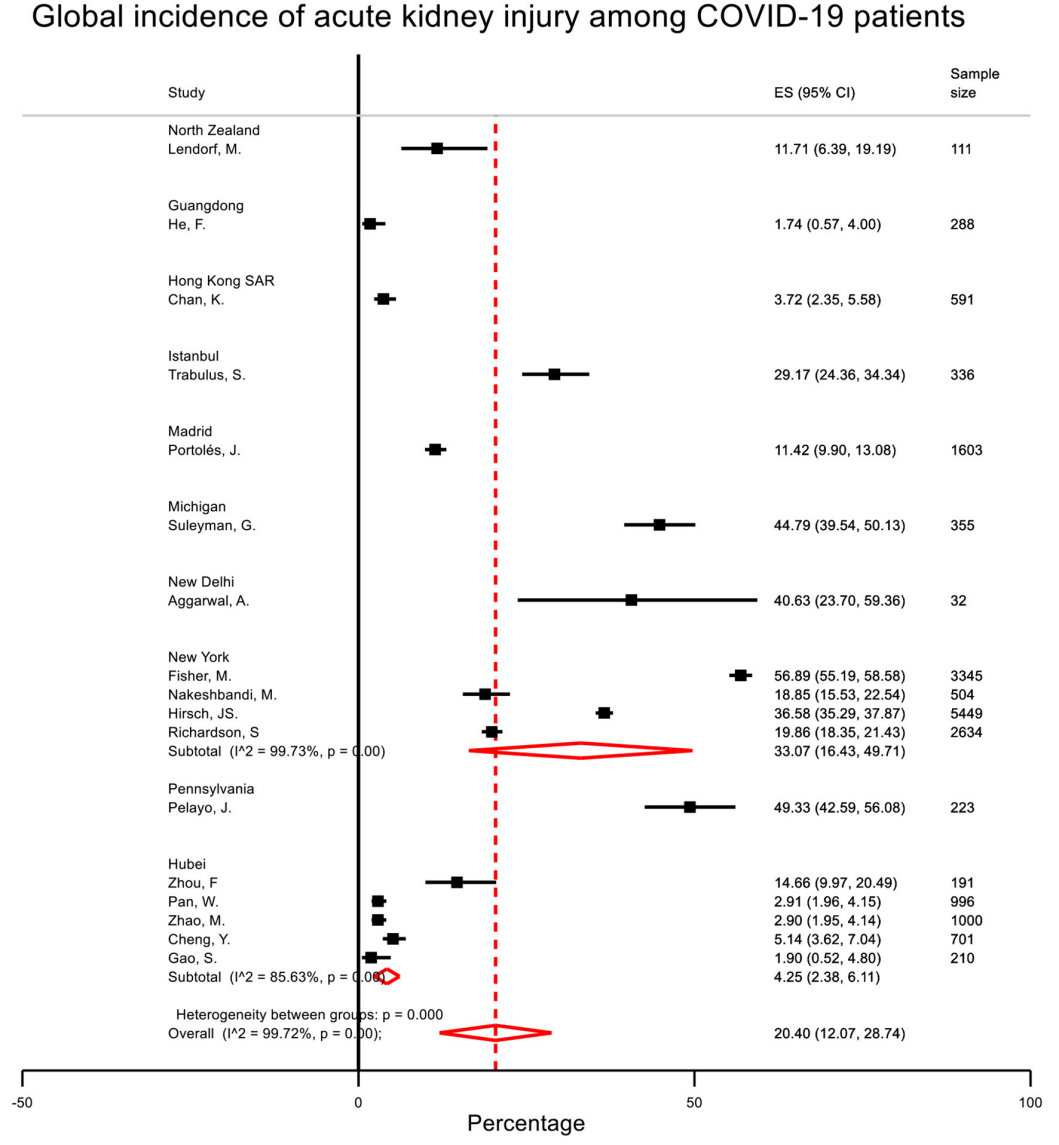
Global incidence of acute kidney injury among COVID-19 patients with no history of renal replacement therapy. The pooled incidence of acute kidney injury (17 studies, *n* = 18,569) was 20.40% with considerable heterogeneity across provinces/states.

After deduplication, 17 studies (*n* = 18,569) reported the incidence of AKI and RRT with clear definition (Table 1, Figure 2, Supplementary 5). The pooled incidence rates of AKI and RRT were 20.40% (95% CI = 12.07–28.74) and 2.97% (95% CI = 1.91–4.04). There was considerable heterogeneity across provinces/states. The incidence rates of AKI in Guangdong, Hong Kong, Hubei, Istanbul, Madrid, Michigan, New Delhi, New York, North Zealand, and Pennsylvania were 1.74, 3.72, 4.25, 29.17, 11.42, 44.79, 40.63, 33.07, 11.71, and 49.33%, respectively. Five studies (*n* = 11,130) reported the prevalence of proteinuria, and three studies (*n* = 7,753) reported the prevalence of hematuria. The prevalence rates of proteinuria and hematuria were 52.09% (95% CI = 34.82–69.37) and 45.38% (95% CI = 27.46–63.31), respectively.

**Table 1:** Global renal manifestations of COVID-19 patients.

**Renal manifestations**	**COVID-19 patients from the general population**	**COVID-19 patients with kidney transplant history**	**Pediatric COVID-19 patients**
Incident acute kidney injury	20.40% (12.07–28.74) 17 studies, *n* = 18,569	35.99% (26.20–45.79) four studies, *n* = 180	16.11% (5.14–27.08) two studies, *n* = 43
Need of renal replacement therapy	2.97% (1.91–4.04) 15 studies, *n* = 12,966	12.65% (0.72–24.58) four studies, *n* = 180	5.54% (−1.14 to 12.21) two studies, *n* = 43
Prevalence of proteinuria	52.09% (34.82–69.37) five studies, n = 11,130	N/A	N/A
Prevalence of hematuria	45.38% (27.46–63.31) three studies, *n* = 7,753	N/A	N/A

*Patients with renal replacement therapy (RRT) history were excluded. The pooled incidence of acute kidney injury (AKI) and RRT (17 studies, n = 18,569) were 20.40 and 2.97%, respectively, with considerable heterogeneity across provinces/states. The prevalence of proteinuria (five studies, n = 11,130) and hematuria (three studies, n = 7,753) were 52.09 and 45.38%, respectively. Although patients who had a transplant presented with a higher incidence of AKI and RRT, their odds of death among AKI patients were lower*.

### Association Between Renal Manifestations and Key Clinical Outcomes

**Figure 3:**
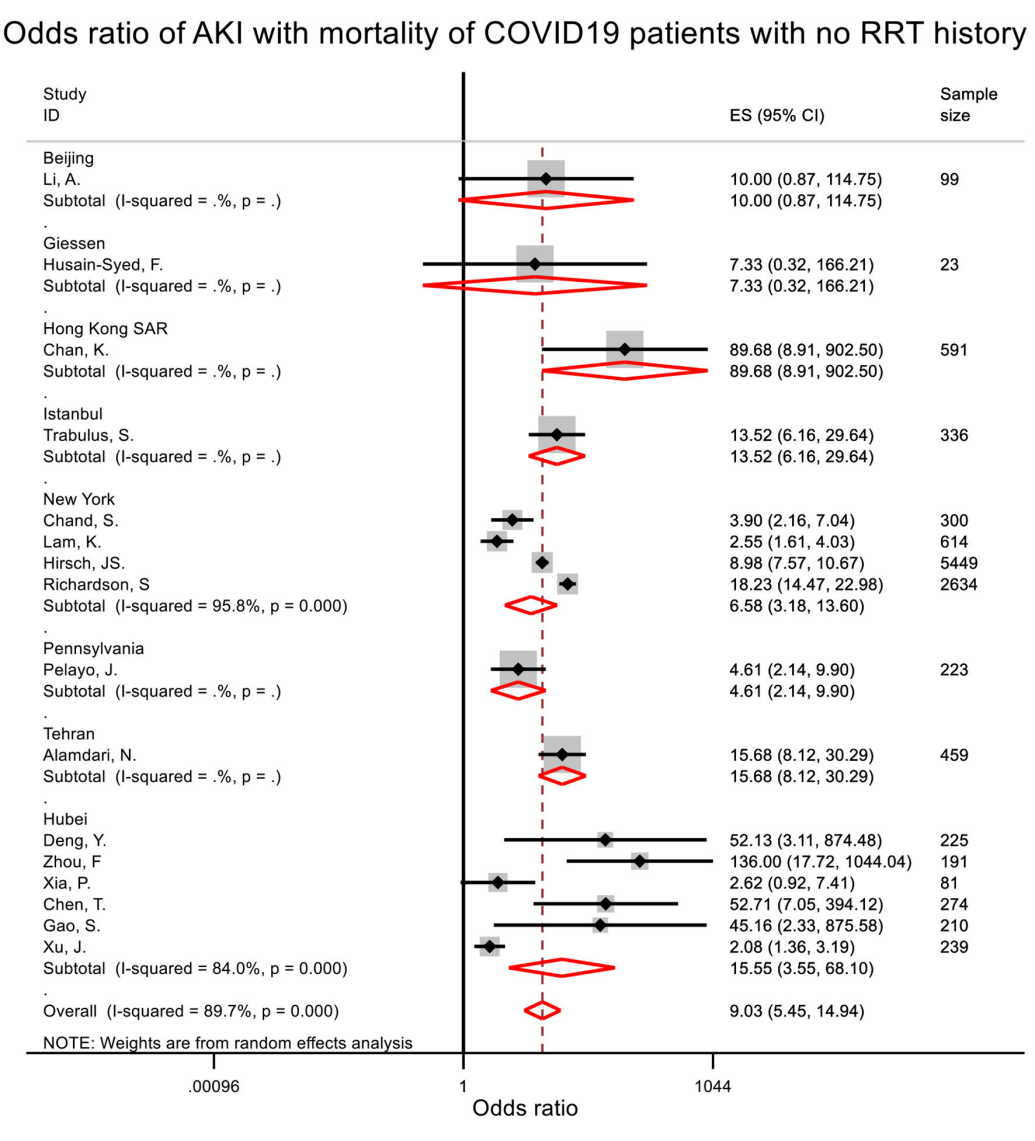
Odds ratio of acute kidney injury with mortality among patients with no renal replacement therapy history. Patients who developed AKI during hospitalization were associated with 8-times increased odds of death.

From the pooled analysis, patients who developed AKI during hospitalization were associated with 8 times (pooled OR = 9.03, 95% CI = 5.45–14.94) and 16.6 times (pooled OR = 17.58, 95% CI = 10.51–29.38) increased odds of death or being critical (Table 2, Figure 3, Supplementary 6). Start of RRT was associated with 18.7 times (pooled OR = 19.69, 95% CI = 4.53–85.70) increased odds of death and 34.0 times (pooled OR = 34.98, 95% CI = 15.17–80.68) increased odds of critical presentation (Table 2, Supplementary 7). AKI stages 1, 2, and 3 were associated with 6.5-, 23.6-, and 93.8-times increased odds of death when compared to patients who did not develop AKI (Table 2).

**Table 2:** Prognosis associated with renal manifestations.

	**Mortality—pooled odds ratio % (95% CI)/*n***	**Critical presentation—pooled odds ratio % (95% CI)/*n***
Acute kidney injury	9.03 (5.45–14.94) 16 studies**/**11,948 patients	17.58 (10.51–29.38) 10 studies**/**7,934 patients
Stage 1	7.45 (2.98–18.67) three studies**/**6,373 patients	N/A
Stage 2	24.64 (2.37–255.78) two studies**/**5,782 patients	N/A
Stage 3	94.77 (10.25–876.37) three studies**/**6,373 patients	N/A
Renal replacement therapy	19.69 (4.53–85.70) 10 studies**/**4,563 patients	34.98 (15.17–80.68) 10 studies**/**18,437 patients

*Patients with renal replacement therapy history were excluded. Pooled odds ratio (by random effect model) of mortality and critical presentation with acute kidney injury. Critical presentation was defined as intensive care admission*.

### Factors Associated With Renal Manifestations

Pooled analysis showed that being older (SMD = 0.57, WMD = 8.39 years); being male (pooled OR = 1.22); with history of diabetes (pooled OR = 2.61), hypertension (pooled OR = 4.07), chronic kidney disease (CKD) (pooled OR = 3.20), or coronary artery disease (pooled OR = 1.68); with higher levels of C-reactive protein (SMD = 0.81, WMD = 22.12 mg/L) and serum creatinine (SMD = 0.75, WMD = 33.79 μmol/L); and lower levels of serum albumin (SMD = −0.37, WMD = −1.91 g/L) at admission were associated with in-hospital AKI (Table 3).

**Table 3:** Factors associated with acute kidney injury worldwide.

**Factor**	**Pooled odds ratio (OR)/weighted mean difference (WMD)/standardized mean difference (SMD) random effects (95% CI)**	**Pooled odds ratio (OR)/standardized mean difference (SMD) fixed effects (95% CI)/n**	**Meta-regression Regression coefficient*^a^* (95% CI) *p*-value*^b^***
Age (years)	WMD = 8.39 (6.01 to 10.77) SMD = 0.57 (0.39 to 0.74) six studies**/**6,400 patients	SMD = 0.48 (0.43 to 0.53) six studies**/**6,400 patients	0.01 (−0.00 to 0.02) *p* = 0.107, 21 studies**/**16,787 patients
Gender (male)	OR = 1.22 (1.09 to 1.36) six studies**/**6,400 patients	OR = 1.22 (1.09 to 1.36) six studies**/**6,400 patients	0.57 (−0.14 to 1.28)^c^ *p* = 0.10, 26 studies**/**20,039 patients
Body mass index (kg/m^2^)	WMD = −0.08 (−2.01 to 1.85) SMD = 0.01 (−0.21 to 0.24) three studies**/**5,695 patients	SMD = 0.07 (0.02 to 0.13) three studies**/**5,695 patients	N/A
**Known comorbidities**			
Diabetes	OR = 2.61 (1.53 to 4.46) six studies**/**6,400 patients	OR = 1.88 (1.68 to 2.10) six studies**/**6,400 patients	0.82 (0.40 to 1.24)^c^ *p* < 0.01, 24 studies**/**18,546 patients
Hypertension	OR = 4.07 (1.80 to 9.19) six studies**/**6,400 patients	OR = 1.90 (1.70 to 2.12) six studies**/**6,400 patients	0.48 (0.18 to 0.78) *p* < 0.01, 25 studies**/**13,062 patients
Chronic kidney disease	OR = 3.20 (1.53 to 6.65) three studies**/**951 patients	OR = 3.36 (1.86 to 6.07) three studies**/**951 patients	0.99 (0.18 to 1.79) *p* = 0.03, 19 studies**/**12,395 patients
Coronary artery disease	OR = 1.68 (1.43 to 1.98) six studies**/**6,400 patients	OR = 1.68 (1.43 to 1.98) six studies**/**6,400 patients	0.63 (−0.68 to 1.93) *p* = 0.335, 15 studies**/**12,442 patients
Tumor	OR = 1.88 (0.54 to 6.52) two studies**/**6,040 patients	OR = 1.24 (0.99 to 1.55) two studies**/**6,040 patients	2.85 (0.93 to 4.76) *p* < 0.01, 18 studies**/**12,895 patients
Time from symptom onset to admission (days)	WMD = −1.99 (−6.12 to 2.14) SMD = −0.32 (−0.98 to 0.34) three studies**/**695 patients	SMD = −0.21 (−0.50 to 0.08) three studies**/**695 patients	0.00 (−0.04 to 0.04) *p* = 0.36, 10 studies**/**3,822 patients
**Laboratory investigations**			
Hemoglobin (g/dL)	WMD = −1.48 (−9.45 to 6.49) SMD = −0.06 (−0.46 to 0.33) three studies**/**695 patients	SMD = −0.07 (−0.36 to 0.21) three studies**/**695 patients	
Leukocyte (10^9^/L)	WMD = 0.84 (−0.55 to 2.23) SMD = 0.28 (−0.13 to 0.88) four studies**/**728 patients	SMD = 0.23 (−0.04 to 0.51) four studies**/**728 patients	
Lymphocytes (10^9^/L)	WMD = −0.16 (−0.28 to −0.04) SMD = −0.15 (−0.55 to 0.25) four studies**/**728 patients	SMD = −0.24 (−0.51 to 0.03) four studies**/**728 patients	
C-reactive protein (mg/L)	WMD = 22.12 (8.08 to 36.15) SMD = 0.81 (−0.14 to 1.76) four studies**/**870 patients	SMD = 0.74 (0.53 to 0.96) four studies**/**870 patients	
Lactate dehydrogenase (U/L)	WMD = 54.13 (17.71 to 90.55) SMD = 0.57 (−0.09 to 1.24) four studies**/**870 patients	SMD = 0.61 (0.34 to 0.89) four studies**/**870 patients	
Serum creatinine (μmol/L)	WMD = 33.79 (12.12 to 55.47) SMD = 0.75 (0.36 to 1.13) five studies**/**6,117 patients	SMD = 0.77 (0.71 to 0.82) five studies**/**6,117 patients	
Serum albumin (g/L)	WMD = −1.91 (−3.81 to −0.00) SMD = −0.37 (−0.71 to −0.02) four studies**/**728 patients	SMD = −0.37 (−0.64 to −0.09) four studies**/**728 patients	

*Patients with renal replacement therapy history were excluded*.

a *By meta-regression with percentage of in-hospital acute kidney injury as dependent variable*,

b *By Monte Carlo test with 3,000 permutations*.

c *The percentage increase in in-hospital AKI rate for every percentage increase in categorical variables. Every percentage increase in the population prevalence of underlying diabetes, hypertension, and chronic kidney disease were associated with 0.82, 0.48, and 0.99% increase in in-hospital AKI, respectively*.

**Figure 4:**
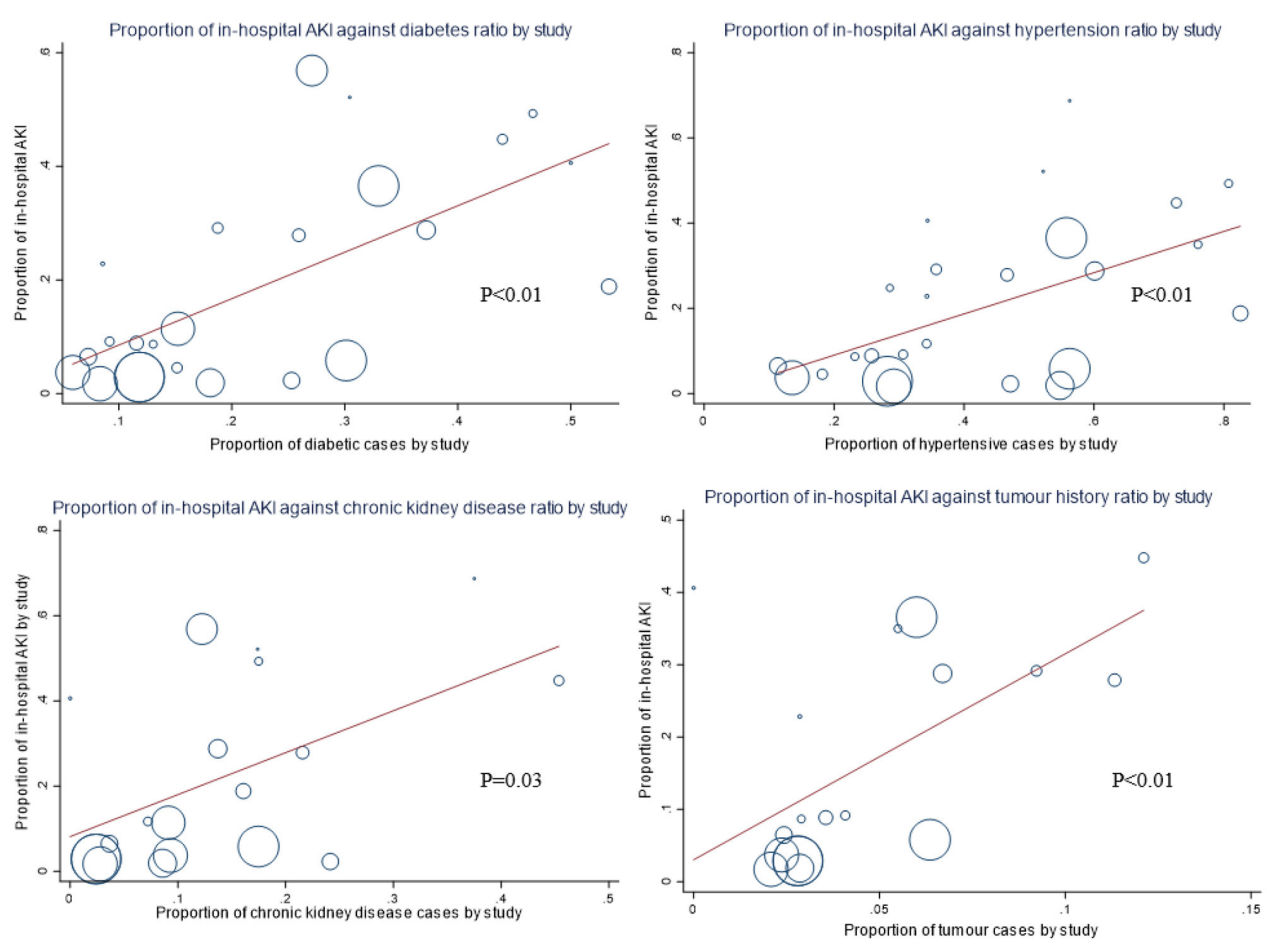
Underlying prevalence of chronic diseases and incident acute kidney injury during COVID-19. Each percentage increase in the population prevalence of underlying diabetes, hypertension, chronic kidney disease, and tumor history was associated with 0.82, 0.48, 0.99, and 2.85% increase in incident in-hospital acute kidney injury (AKI), respectively.

**Figure 5:**
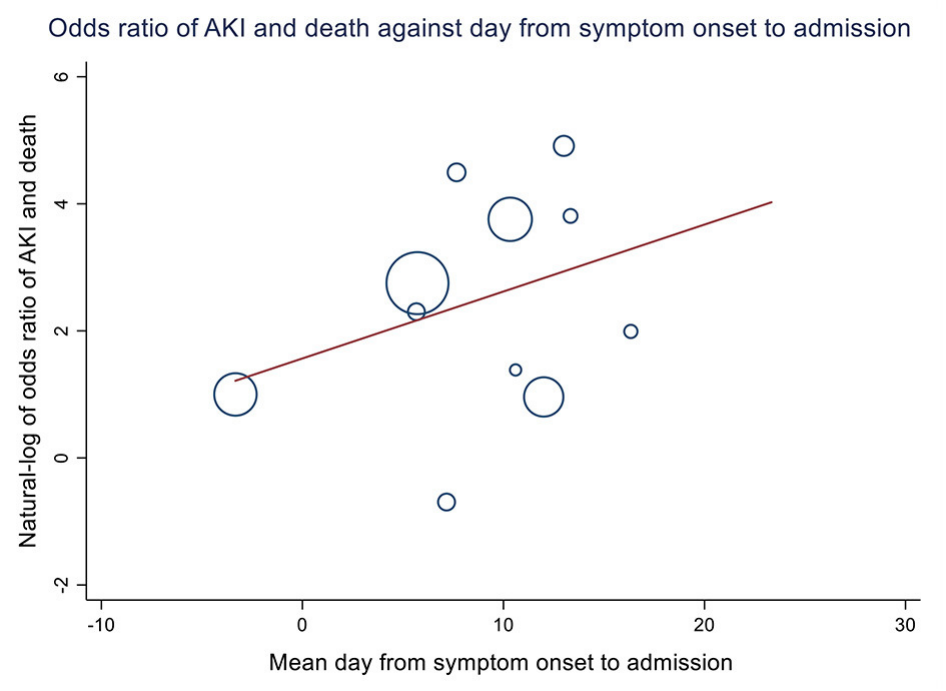
Association between days from symptom onset to admission and odds ratio of death and acute kidney injury. Eleven studies from China, Italy, Spain, Germany, and Turkey were included (2,097 patients). Each day longer between symptom onset and admission was associated with a 12% increase in the odds of death among acute kidney injury (AKI) patients across population, although not statistically significant (OR = 1.12, 95% CI = 0.90–1.38, *p* = 0.314).

From the meta-regression of studies with consecutive or random sampling from different clinical settings, every percentage increase in the population prevalence of underlying diabetes, hypertension, chronic kidney disease, and tumor history was associated with 0.82% (95% CI = 0.40–1.24), 0.48% (95% CI = 0.18–0.78), 0.99% (95% CI = 0.18–1.79), and 2.85% (95% CI = 0.93–4.76) increase in incident in-hospital AKI, respectively (Table 3, Figure 4). There was a positive trend of increased odds of death among AKI patients against the interval between symptom onset and admission (Figure 5). Clinically, cohorts with higher percentage of patients who presented with dyspnea (*p* < 0.01), diarrhea (*p* = 0.01), and cough (*p* = 0.04) were associated with a higher incidence of in-hospital AKI (Supplementary 8). A positive trend of AKI incidence with fatigue was observed, but not fever.

### Patients With Kidney Transplant History and Pediatric Patients

From the pooled analysis of four studies (*n* = 180) on COVID-19 patients with kidney transplant history, patients who had a transplant presented with a higher incidence of AKI (35.99% vs. 20.40%) and RRT (12.65% vs. 2.97%) when compared to COVID-19 patients from the general population (Table 1). The odds of death among AKI patients (three studies, *n* = 110) was lower among COVID-19 patients with kidney transplant history when compared to COVID-19 patients from the general population (OR = 2.24 vs. 9.03) (Supplementary 9). The same trend was also observed in the OR of incident RRT and mortality among patients with kidney transplant history (OR = 4.24 vs. 19.69). Three studies on pediatric COVID-19 patients were identified, and two studies (*n* = 43) reported consecutive or random sampling. The mean age was 8.9 ± 1.27 years. The incidence rates of in-hospital AKI and RRT were 16.11 and 5.54%, respectively.

## Discussion

Our findings showed that the underlying prevalence of diabetes, hypertension, chronic kidney disease, and tumor history was associated with increased incidence of AKI at population level across different settings. A positive trend of increased odds of death among AKI patients against the interval between symptom onset and admission was observed. Although patients who had a transplant presented with a higher incidence of AKI and RRT, the odds of death among AKI patients was lower.

### Public Health Implication—Underlying Comorbidities and Future Health Services Need

The meta-regression analysis demonstrated that the underlying prevalence of non-communicable diseases including diabetes, hypertension, chronic kidney disease, and tumor history varied across different settings. A recent modeling study also demonstrated that underlying non-communicable diseases were associated with increased severity of COVID-19 (31). We further quantified the effect of various common chronic conditions on the incidence of AKI. The differential contribution of underlying non-communicable diseases in different cohorts partly explained the heterogeneity of the observed in-hospital AKI incidence. Our results suggested that poor control of non-communicable diseases at the population level was associated with increased burden of AKI and RRT and the associated critical presentation and mortality during the COVID-19 outbreak.

Globally, 20.40 and 2.97% of patients developed in-hospital AKI and required RRT. Incident AKI and RRT were associated with increased risk of developing chronic kidney disease, end-stage kidney disease (15), diabetes (16), hypertension (17), and recurrent AKI (32). Our analysis on the Hong Kong cohort also showed a sustained reduced renal function after 6 months among COVID-19 patients with in-hospital AKI (2). Incident end-stage kidney disease due to residual kidney impairment is anticipated to increase the need of long-term renal service including dialysis substantially, which could be costly and limited (33, 34).

### Clinical Implication—Length of Delayed Admission, Odds of Death, and Community Isolation Facilities

There was considerable variance in the odds of AKI and mortality across different studies. We observed a trend of positive association between the time from symptom onset to admission and the odds of death of AKI, although not statistically significant because of the limited number of studies with relevant data. A recent retrospective cohort study showed that higher viral load at admission was associated with increased in-hospital AKI (35). We hypothesize that delayed admission would lead to an increased viral load at admission and therefore increased incidence and severity of AKI. Besides, our previous analysis on the Hong Kong cohort showed that the incidence of in-hospital AKI was comparable among patients who were symptomatic and asymptomatic at admission (2). As most jurisdictions only tested and admitted patients who were symptomatic, there was a possible delay in identifying AKI before patients were admitted. Early identification of abnormal fluid balance is important to the prevention and management of AKI, which could be corrected (36). Selective admission of symptomatic patients may lead to an overall lengthened latent period between disease onset and admission and could be associated with increased mortality after AKI.

As the inpatient service load is expected to reduce after vaccination program is rolled out, admitting patients with milder symptoms or using community isolation facilities to provide basic screening and care for mild or asymptomatic cases could help to identify AKI earlier for treatment (2, 37) and further mitigate transmission (38). Nevertheless, more studies are needed to evaluate the effect of early admission and commencement of antiviral treatment on the incidence of AKI, RRT, and mortality.

### Lower Mortality Amid Higher AKI Incidence Among Kidney Transplant Patients

From the subgroup analysis, patients with kidney transplant history had a higher incidence of AKI but unexpectedly lower mortality when compared to those without kidney transplant history. In the studies that contributed to the meta-regression, cyclosporine, mycophenolic mofetil, tacrolimus, dexamethasone, and prednisone were the commonly used medications among the kidney transplant patients (39–41).

Cytokine storm and acute respiratory distress syndrome were strongly correlated with mortality among COVID-19 patients (42). In a previous retrospective cohort of transplant patients receiving immunosuppressive therapy, COVID-19 patients who took cyclosporine generally presented with mild symptoms (43). The COQUIMA cohort from Spain with 607 patients showed that the use of cyclosporine was associated with 76% reduction in mortality, the strongest among other therapies such as antivirals (44). Besides, a previous systematic review showed that mycophenolic mofetil inhibits severe acute respiratory syndrome coronavirus 2 (SARS-CoV-2) replication *in vitro* (45). Currently, there are only four clinical trials listed on Clinicaltrials.gov evaluating the effectiveness of cyclosporine on COVID-19, and one open-label randomized controlled trial in Spain has started recruitment (46).

The use of corticosteroids in the transplant cohorts could indicate (1) high disease severity and (2) more intensive management of the transplant patients. Corticosteroids and intensive immunosuppression, as trial COVID-19 treatment, have been shown to reduce the mortality of severe COVID-19 patients (47, 48). The use of immunosuppressive drugs among COVID-19 patients with kidney transplant did not appear to increase the AKI-associated mortality from our results and other existing evidence.

### Strengths and Limitations

This is the first global meta-analysis to quantify the disease burden of non-communicable diseases on AKI for COVID-19 patients. We avoided overlapping data by removing studies from possible duplicated data source though matching the location, institution, and time period. The included studies in the systematic review were of good quality, and therefore the results from meta-analysis are reliable. In the meta-regression, we were also able to demonstrate a trend of positive correlation between days from symptom onset to admission and odds of death among AKI patients.

However, this study has several limitations. First, we included only studies identified from electronic database and did not search for gray literature. As there is alarming volume of studies produced since the COVID-19 outbreak, the search on gray literature that was not peer-reviewed will substantially increase the length of review process and was not possible because of resource limitation. We minimized the impact by a thorough reference list search to cover major cohorts. Besides, we did not include studies with unclear definition of AKI for the meta-analysis on the AKI-related analyses to ensure the consistency of outcomes. Therefore, several studies were unavoidably excluded from the analyses. Multivariable meta-regression was not performed because of the limited number of studies reporting the underlying communicable diseases and renal manifestations. Also, the subgroups of pediatric patients and kidney transplant patients were small, and therefore more in-depth regression analysis was not performed, including the assessment of drug effect on mortality. Lastly, the specific effect of SARS-CoV-2 with different genetic subtype markers (49) on renal manifestation is not assessable as related data are unavailable. Previous study demonstrated that eight variants of SARS-CoV-2 were associated with higher mortality, and the renal manifestations of different variants would require further investigation (50).

## Conclusion

By meta-regression, the heterogeneity in global AKI incidence at population level could be explained by the underlying prevalence of non-communicable diseases including diabetes, hypertension, chronic kidney disease, and tumor history. Delay in admission after symptom onset demonstrated a positive trend with higher mortality among AKI patients. From the observation of lower mortality among kidney transplant patients, further clinical assessment on the effect of immunosuppressants, in particular cyclosporine, on COVID-19 patients is warranted.

## Existing Evidence

Acute kidney injury (AKI) is common, critical, and associated with in-hospital mortality and a series of chronic complications. Earlier studies showed that existing non-communicable diseases were correlated with coronavirus disease 2019 (COVID-19) severity. However, the magnitude of effect of underlying non-communicable diseases on COVID-19–related AKI and the factors affecting the odds of death among AKI patients remain unclear.

## Added Value

1. In this meta-analysis of 74 studies, global AKI and renal replacement therapy (RRT) incidence of COVID-19 patients were 20.40 and 2.97% among patients with no RRT history. Prevalence rates of proteinuria and hematuria were 52.09 and 45.38%.
2. At population level, each percentage increase in the underlying prevalence of diabetes, hypertension, chronic kidney disease, and tumor history was associated with 0.82, 0.48, 0.99, and 2.85% increased incidence of AKI across different settings, respectively.
3. Although patients who had a kidney transplant presented with a higher incidence of AKI and RRT, their odds of death among AKI patients was lower.
4. A positive trend of increased odds of death among AKI patients against the interval between symptom onset and hospital admission was observed.

## Implication

Underlying prevalence of non-communicable diseases partly explained the heterogeneity in AKI incidence globally. Delay in admission could be associated with higher mortality among AKI patients.

## Data Availability Statement

The original contributions presented in the study are included in the article/Supplementary Material, further inquiries can be directed to the corresponding author/s.

## Author Contributions

KC and SC-WT conceived the study and drafted the manuscript. KC retrieved articles from database and performed the statistical analyses. PL and KY screened the articles. KC and KY performed data extraction and synthesis. PL and KC assessed the study quality. All authors proofread the manuscript.

## Conflict of Interest

The authors declare that the research was conducted in the absence of any commercial or financial relationships that could be construed as a potential conflict of interest.

## Abbreviations

AKI: acute kidney injury
COVID-19: coronavirus disease 2019
KDIGO: Kidney Disease Improving Global Outcomes
OR: odds ratio
RRT: renal replacement therapy
SMD: standardized mean difference
SARS-CoV-2: severe acute respiratory syndrome coronavirus 2
WMD: weighted mean difference.
