# Supplementary material for: Global REnal Involvement of CORonavirus Disease 2019 (RECORD): A Systematic Review and Meta-Analysis of Incidence, Risk Factors, and Clinical Outcomes
Source: Front Med (Lausanne). 2021 May 25;8:678200. doi: 10.3389/fmed.2021.678200 (PMC8185046; doi:10.3389/fmed.2021.678200)
Supplement: Supplementary file 1 [file Data_Sheet_1.docx]

**Global REnal involvement of CORonavirus Disease 2019 (RECORD):**

**A systematic review and meta-analysis of incidence, risk factors and clinical outcomes Supplementary File**

| S1 Search strategy of the systematic review | P. 2 |
| --- | --- |
| S2 List of excluded studies of systematic review | P. 5 |
| S3 Characteristics of included studies for meta-analysis | P. 59 |
| S4 Quality assessment of included studies of meta-analysis | P. 80 |
| S5 Global incidence of renal manifestations therapy among COVID-19 patients with no history of renal replacement therapy | P. 100 |
| S6 Odds ratios of acute kidney injury with critical presentation among COVID-19 patients with no history of renal replacement therapy | P. 102 |
| S7 Odds ratios of renal replacement therapy with mortality and critical presentation among COVID-19 patients with no history of renal replacement therapy | P. 103 |
| S8 Association between clinical presentations and odds ratio of acute kidney injury and mortality | P. 105 |
| S9 Odds ratios of acute kidney injury with mortality among COVID-19 patients with history of renal replacement therapy | P. 108 |

**S1 Search strategy of the systematic review**

**Electronic database**

1. **Cochrane (as of 2020.10.5)**

#1 [mh “coronavirus”]

#2 (*COVID*):ti,ab,kw

#3 (*nCoV*):ti,ab,kw

#4 (*coronavir*):ti,ab,kw

#5 (SARS*):ti,ab,kw

#6 #1 OR #2 OR #3 OR #4 OR #5

**Total: 2322**

All limited to Cochrane Reviews, Trials, and Editorials published from Dec 2019 to present.

1. **Ovid MEDLINE(R) and Epub Ahead of Print, In-Process & Other Non-Indexed Citations, Daily and Versions(R) (via Ovid) 1946 to 1 October 2020**
2. **Embase Classic + Embase (via Ovid) 1947 to 1 October 2020**

1. exp Coronavirus/

2. exp Coronavirus Infections/

3. (*Coronavir* or *nCov* or *covid* or *SARS*).ti,ab,kf.

4. 1 or 2 or 3

5. $kidney$.mp. [mp=title, abstract, original title, name of substance word, subject heading word, floating sub-heading word, keyword heading word, organism supplementary concept word, protocol supplementary concept word, rare disease supplementary concept word, unique identifier, synonyms]

6. $renal$.mp. [mp=title, abstract, original title, name of substance word, subject heading word, floating sub-heading word, keyword heading word, organism supplementary concept word, protocol supplementary concept word, rare disease supplementary concept word, unique identifier, synonyms]

7. $glomerul$.mp. [mp=title, abstract, original title, name of substance word, subject heading word, floating sub-heading word, keyword heading word, organism supplementary concept word, protocol supplementary concept word, rare disease supplementary concept word, unique identifier, synonyms]

8. $nephro$.mp. [mp=title, abstract, original title, name of substance word, subject heading word, floating sub-heading word, keyword heading word, organism supplementary concept word, protocol supplementary concept word, rare disease supplementary concept word, unique identifier, synonyms]

9. $AKI$.mp. [mp=title, abstract, original title, name of substance word, subject heading word, floating sub-heading word, keyword heading word, organism supplementary concept word, protocol supplementary concept word, rare disease supplementary concept word, unique identifier, synonyms]

10 $CKD$.mp. [mp=title, abstract, original title, name of substance word, subject heading word, floating sub-heading word, keyword heading word, organism supplementary concept word, protocol supplementary concept word, rare disease supplementary concept word, unique identifier, synonyms]

11 $ESRD$.mp. [mp=title, abstract, original title, name of substance word, subject heading word, floating sub-heading word, keyword heading word, organism supplementary concept word, protocol supplementary concept word, rare disease supplementary concept word, unique identifier, synonyms]

12 $ESKD$.mp. [mp=title, abstract, original title, name of substance word, subject heading word, floating sub-heading word, keyword heading word, organism supplementary concept word, protocol supplementary concept word, rare disease supplementary concept word, unique identifier, synonyms]

13. 5 or 6 or 7 or 8 or 9 or 10 or 11 or 12

14. 4 and 13

15. Human/

16. 14 and 15

17. limit 16 to yr="2020"

**Medline total: 798**

**Embase total: 359**

1. **PubMed (from 1 Dec 2019 onwards)**

(((((((((kidney*) OR renal*) OR nephro*) OR AKI*) OR CKD*) OR ESRD*) OR ERKD*)) AND (((((SARS*) OR nCov*) OR Coronavir*) OR COVID*) OR coronavirus[MeSH Terms])) AND ( ( "2019/12/01"[PDat] : "3000/12/31"[PDat] ) AND Humans[Mesh]))

**PubMed total: 1156**

1. **China National knowledge Infrastructure 中國期刊全文數據庫 (CNKI)**

((SU=冠状病毒 or KY=冠状病毒 or TI=冠状病毒) or (SU=新型冠状病毒 or KY=新型冠状病毒 or TI=新型冠状病毒) or (SU=2019‐nCoV or KY=2019‐nCoV or TI=2019‐nCoV)) AND (FT=肾) AND (PT=2020)

**CNKI total: 1864**

1. **Wanfang Med Online**[**万方数据. 中国学术会议文献数据库　(医药卫生**](https://julac.hosted.exlibrisgroup.com/primo-explore/fulldisplay?docid=HKU_IZ61495237710003414&context=L&vid=hku_er&lang=en_US&tab=er&query=any,contains,wanfang&sortby=rank&facet=rtype,include,Databases&offset=0)**)**

((((摘要=冠状病毒) OR 题名=冠状病毒)) OR ((摘要=新型冠状病毒) OR 题名=新型冠状病毒))) AND (肾)

Limit: 出版时间=2019-2020**Total: 70**

1. **Reference list search: 8**

**S2 List of excluded studies of systematic review**

| **ID** | **First Author** | **Title** | **Reason^** | **Remarks** |
| --- | --- | --- | --- | --- |
| 2 | 丁玉曦 | 新型冠狀病毒肺炎（COVID-19）研究現狀 | 5 | Narrative review with no data collection on renal manifestations |
| 3 | 于洪志 | 天津地區新型冠狀病毒肺炎患者臨床特征分析 | 5 | No data collection on renal manifestations |
| 4 | 伍偉 | 新型冠狀病毒肺炎102例臨床特征分析 | 5 | No data collection on renal manifestations |
| 5 | 何穎雪 | 新型冠狀病毒肺炎患者伴肝功能受損的臨床特征分析 | 5 | No data collection on renal manifestations |
| 6 | 何虹 | 維持性血液透析患者合并新型冠狀病毒肺炎2例報告 | 5 | Case report with no additional quantitative data on renal manifestations under investigation |
| 7 | 余增淵 | 新生兒重型新型冠狀病毒肺炎轉運治療1例病例報告 | 5 | Case report with no data collection on renal manifestations |
| 8 | 余思邈 | 25例新型冠狀病毒肺炎患者臨床特征分析 | 5 | Retrospective cohort with reporting on renal manifestations |
| 9 | 侯可可 | 新型冠狀病毒肺炎不同時期CT表現及中性粒細胞/淋巴細胞比值、T淋巴細胞亞群變化 | 5 | Retrospective cohort with no data collection on renal manifestations |
| 10 | 侯寧寧 | 新型冠狀病毒（SARS-CoV-2）的起源和檢測方法 | 5 | Narrative review with no data collection on renal manifestations |
| 11 | 先小乐 | 老年慢性肾衰竭合并新型冠状病毒肺炎中医防治思路初探 | 5 | Narrative review |
| 12 | 凌學斌 | 457例新型冠狀病毒肺炎輕癥患者的臨床及CT特征 | 5 | No data collection on renal manifestations |
| 13 | 劉利容 | 商丘市91例新型冠狀病毒肺炎流行病學特征分析 | 5 | No data collection on renal manifestations |
| 14 | 劉勛 | 湖北省新型冠狀病毒肺炎疫情的時空分布特征初步分析 | 5 | No data collection on renal manifestations |
| 15 | 劉映霞 | 新型冠狀病毒(2019-nCoV)感染患者肺損傷相關的臨床及生化指標研究 | 5 | Retrospective cohort with no quantitative data of renal manifestations |
| 16 | 劉松 | 新型冠狀病毒肺炎患者的臨床特點及肺部CT變化模式 | 5 | Retrospective cohort with no data collection on renal manifestations |
| 17 | 劉海艇 | 2019新型冠狀病毒肺炎腎功能及尿常規指標分析 | 5 | No data collection on renal manifestations |
| 18 | 劉澗 | 新型冠狀病毒肺炎（普通型）與疑似患者臨床特征比較 | 5 | No data collection on renal manifestations |
| 19 | 劉茜 | 新型冠狀病毒肺炎死亡尸體系統解剖大體觀察報告 | 5 | Autopsy reports |
| 20 | 劉雯雯 | 兒童SARS、MERS和COVID-19的流行病學和臨床特征 | 5 | Narrative review with no data collection on renal manifestations |
| 21 | 史河水 | 新型冠狀病毒(2019-nCoV)感染的肺炎臨床特征及影像學表現 | 5 | Narrative review with no data collection on renal manifestations |
| 22 | 吉攀 | 新型冠狀病毒肺炎重癥患者的臨床特征及病情嚴重程度的相關因素 | 5 | No data collection on renal manifestations |
| 23 | 向天新 | 江西地區49例新型冠狀病毒肺炎患者臨床特征分析 | 5 | Retrospective cohort with no data collection on renal manifestations |
| 24 | 呂耀東 | 新型冠狀病毒肺炎患者的臨床特征及治療轉歸分析 | 5 | No data collection on renal manifestations |
| 25 | 周婧 | 新型冠狀病毒導致多器官功能衰竭的機制探討 | 5 | Narrative review with no data collection on renal manifestations |
| 26 | 周玉平 | 咸寧地區3886例發熱、咳嗽患者臨床特征與實驗室檢查結果分析 | 5 | No data collection on renal manifestations |
| 27 | 周玉平 | 新型冠狀病毒肺炎患者相關血液檢測指標分析 | 5 | No data collection on renal manifestations |
| 28 | 周耿標 | 新型冠狀病毒肺炎患者舌象特征與臨床分型的關系 | 5 | No data collection on renal manifestations |
| 29 | 周花萍 | 霧化吸入IFN-α治療病毒性疾病的研究進展 | 5 | Narrative review with no data collection on renal manifestations |
| 30 | 周靜 | 安徽省144例新型冠狀病毒肺炎患者中醫證候特征 | 5 | No data collection on renal manifestations |
| 31 | 唐子健 | 從2019-nCoV和SARS-CoV的侵襲靶點血管緊張素轉化酶2尋找新型冠狀病毒肺炎的救治策略 | 5 | Narrative review with no data collection on renal manifestations |
| 32 | 唐德志 | 從調節“腎精”狀態淺談老年人新型冠狀病毒肺炎的防治 | 5 | Narrative review with no data collection on renal manifestations |
| 33 | 唐鋒 | 2019-2020年冬春季武漢兒童醫院收治流感和新型冠狀病毒肺炎患兒的流行病學特點 | 5 | No data collection on renal manifestations |
| 34 | 夏文廣 | 中西醫結合治療新型冠狀病毒肺炎34例臨床研究 | 5 | RCT with no reporting on renal manifestations |
| 35 | 夏露 | 中西醫結合治療新型冠狀病毒肺炎100例療效及肝損傷情況分析 | 5 | No data collection on renal manifestations |
| 36 | 孔令喜 | 新型冠狀病毒肺炎肝損傷特征分析 | 5 | No data collection on renal manifestations |
| 37 | 孟憲澤 | 新型冠狀病毒肺炎患者中醫證候756例分析 | 5 | No data collection on renal manifestations |
| 38 | 季燁龍 | 新型冠狀病毒肺炎患者合并糖尿病的臨床特點及預后 | 5 | No data collection on renal manifestations |
| 39 | 孫建芳 | 102例武漢火神山醫院新型冠狀病毒肺炎患者的臨床特征分析 | 5 | No data collection on renal manifestations |
| 40 | 宋杲 | 新型冠狀病毒（2019-nCoV）治療藥物體內外研究及藥物研發進展 | 5 | Narrative review with no data collection on renal manifestations |
| 41 | 宗陽 | 以血管緊張素轉換酶Ⅱ(ACE2）為受體挖掘治療新型冠狀病毒肺炎（COVID-19）潛在中藥及單體成分 | 5 | In silico |
| 42 | 尚偉鋒 | 新型冠狀病毒肺炎臨床特征的meta分析 | 5 | Systematic review with no quantitative data on renal manifestations |
| 43 | 岳麗娜 | 150例新型冠狀病毒肺炎CT影像表現分析 | 5 | No data collection on renal manifestations |
| 44 | 崔小萌 | 新型冠狀病毒肺炎患者治療前后臨床特征分析——一項單中心的回顧性研究 | 5 | Narrative review with no data collection on renal manifestations |
| 45 | 巴元明 | “肺炎1號”治療新型冠狀病毒肺炎451例多中心臨床研究 | 5 | No data collection on renal manifestations |
| 46 | 張兵華 | 老年新型冠狀病毒肺炎病人臨床特征及死亡風險相關因素分析 | 5 | No data collection on renal manifestations |
| 47 | 張凡 | 451例新型冠狀病毒感染患者合并肝損傷的發生情況及臨床特征分析 | 5 | No data collection on renal manifestations |
| 48 | 張國英 | 新型冠狀病毒肺炎合并肝功能異常1例病例報告 | 5 | Case report with no additional quantitative data on renal manifestations under investigation |
| 49 | 張婧 | 廣州市241例新型冠狀病毒肺炎患者的抗病毒治療用藥分析 | 5 | No data collection on renal manifestations |
| 50 | 張昕 | 新型冠狀病毒感染的流行特點與趨勢 | 5 | Narrative review with no data collection on renal manifestations |
| 51 | 張磊 | 新型冠狀病毒肺炎嚴重程度危險因素的分析 | 5 | No data collection on renal manifestations |
| 53 | 張騰飛 | 新型冠狀病毒肺炎患者尿蛋白與病程的相關性 | 5 | No data collection on renal manifestations |
| 55 | 彭俊男 | 重慶市新型冠狀病毒肺炎患者并發急性呼吸窘迫綜合征風險預測模型的建立 | 5 | No data collection on renal manifestations |
| 56 | 徐曉涵 | 新型冠狀病毒肺炎治療中抗病毒藥物的合理使用 | 5 | Narrative review with no data collection on renal manifestations |
| 57 | 戢太陽 | 4例兒童新型冠狀病毒肺炎的臨床特點和藥物治療分析 | 5 | Case report with no additional quantitative data on renal manifestations under investigation |
| 58 | 戴威 | 輕型新型冠狀病毒肺炎患者臨床特征分析 | 5 | No data collection on renal manifestations |
| 59 | 戴敏 | 嶺南新型冠狀病毒肺炎臨床表現的初步分析 | 5 | No data collection on renal manifestations |
| 60 | 房曉偉 | 2019新型冠狀病毒感染的肺炎79例臨床特征及治療分析 | 5 | Retrospective cohort with no data collection on renal manifestations |
| 61 | 文玉先 | COVID-19疫情期間腎內科住院患者管理實踐 | 5 | Narrative review with no data collection on renal manifestations |
| 62 | 方琦璐 | 查爾酮及其衍生物防治新型冠狀病毒肺炎（COVID-2019）潛在應用的研究進展 | 5 | Narrative review with no data collection on renal manifestations |
| 63 | 時佳 | 中西醫結合治療上海地區49例非危重型新型冠狀病毒肺炎臨床療效觀察 | 5 | RCT with no reporting on renal manifestations |
| 64 | 曹愛華 | 山東省兒童新型冠狀病毒感染者流行病學及臨床特征 | 5 | No data collection on renal manifestations |
| 65 | 李丹 | 株洲地區80例新型冠狀病毒肺炎患者臨床特征分析 | 5 | Retrospective cohort with no data collection on renal manifestations |
| 66 | 李偉男 | 辨證論治在105例新型冠狀病毒肺炎中西醫結合診治中的作用 | 5 | No data collection on renal manifestations |
| 67 | 李天志 | 新型冠狀病毒肺炎診治研究進展 | 5 | Narrative review with no data collection on renal manifestations |
| 68 | 李建生 | 河南省524例新型冠狀病毒肺炎患者臨床特征及中醫證候分布 | 5 | No data collection on renal manifestations |
| 70 | 李昀澤 | 清肺飲治療新型冠狀病毒肺炎合并高血壓病患者152例臨床療效分析 | 5 | No data collection on renal manifestations |
| 71 | 李昊 | 2019冠狀病毒病749例患者中醫藥治療的真實世界臨床研究 | 5 | No data collection on renal manifestations |
| 72 | 李會敏 | 武漢地區188例新型冠狀病毒肺炎普通型患者發病初期中醫四診信息分析 | 5 | No data collection on renal manifestations |
| 73 | 李瑞云 | 白細胞增高的新型冠狀病毒肺炎患者的臨床特征 | 5 | No data collection on renal manifestations |
| 74 | 李興超 | 山東省新型冠狀病毒肺炎老年病例的流行病學特征 | 5 | No data collection on renal manifestations |
| 75 | 李若青 | 新型冠狀病毒肺炎重癥患者相關危險因素多中心臨床研究 | 5 | No data collection on renal manifestations |
| 76 | 李長力 | 鄂地輸入型和本地繼發型新型冠狀病毒肺炎的臨床特點研究 | 5 | No data collection on renal manifestations |
| 77 | 林政峰 | 205例新型冠狀病毒肺炎病例臨床分析 | 5 | No data collection on renal manifestations |
| 79 | 梁亞林 | 28例新型冠狀病毒肺炎確診病例流行病學和臨床特征分析 | 5 | Retrospective cohort with no data collection on renal manifestations |
| 80 | 梁珀銘 | 武漢市金銀潭醫院139例新型冠狀病毒肺炎患者血清白蛋白水平與病情和預后關聯分析 | 5 | No data collection on renal manifestations |
| 81 | 楊凱 | 57例非疫區COVID-19出院患者流行病學及臨床特點分析 | 5 | Retrospective cohort with no data collection on renal manifestations |
| 82 | 楊勇 | 新型冠狀病毒肺炎治療聯合用藥潛在藥品不良反應分析 | 5 | Narrative review with no data collection on renal manifestations |
| 83 | 楊曉明 | 康復期血漿應用于急性病毒性傳染病現狀及其治療新型冠狀病毒肺炎前景 | 5 | Narrative review with no data collection on renal manifestations |
| 84 | 楊羽君 | 中醫藥治療人類高致病性冠狀病毒SARS-CoV-2與SARS-CoV感染肺炎的思考 | 5 | Narrative review with no data collection on renal manifestations |
| 85 | 楊靜 | 新型冠狀病毒肺炎普通型病例40例臨床研究 | 5 | Retrospective cohort with no data collection on renal manifestations |
| 86 | 汪成 | 新型冠狀病毒肺炎患者心肌損傷的臨床分析 | 5 | No data collection on renal manifestations |
| 87 | 沙廣娟 | 1例兒童感染新型冠狀病毒合并流感病毒的報告 | 5 | Case report with no additional quantitative data on renal manifestations under investigation |
| 88 | 熊鵬 | 新型冠狀病毒肺炎患者660例臨床特征分析 | 5 | No data collection on renal manifestations |
| 89 | 王儒涵 | 襄陽市306例新型冠狀病毒肺炎患者的臨床特征、治療及預后分析 | 5 | No data collection on renal manifestations |
| 90 | 王愛華 | 渝西地區陳舊性心肌梗死患者2019-nCoV知信行調查研究 | 5 | No data collection on renal manifestations |
| 91 | 王琳 | 新型冠狀病毒肺炎患者的治療及不良反應監護 | 5 | Retrospective cohort with no data collection on renal manifestations |
| 92 | 王翅鵬 | 普通型與重型新型冠狀病毒肺炎的HRCT征象對照研究 | 5 | No data collection on renal manifestations |
| 93 | 王苑 | 新型冠狀病毒肺炎病人床邊血液透析的實施與防護 | 5 | Narrative review with no data collection on renal manifestations |
| 94 | 王谷宜 | 2019冠狀病毒病老年患者的臨床特點及重癥的危險因素 | 5 | No data collection on renal manifestations |
| 95 | 盧子龍 | COVID-19患者臨床特征及免疫功能分析 | 5 | No data collection on renal manifestations |
| 96 | 石璞玉 | 新冠肺炎診療關鍵要點總結 | 5 | Narrative review with no data collection on renal manifestations |
| 97 | 秦奇偉 | 江西省215例新型冠狀病毒肺炎病例的實驗室檢查特征 | 5 | No data collection on renal manifestations |
| 98 | 秦紅亞 | 2019新型冠狀病毒肺炎研究進展 | 5 | Narrative review with no data collection on renal manifestations |
| 99 | 程克斌 | 普通型和重型新型冠狀病毒肺炎康復患者463例臨床特征分析 | 5 | No data collection on renal manifestations |
| 100 | 程永慶 | 重組人干擾素α1b與新型冠狀病毒肺炎防治 | 5 | Narrative review with no data collection on renal manifestations |
| 101 | 程芳 | 新型冠狀病毒肺炎普通型和重癥病例臨床特征比較 | 5 | No data collection on renal manifestations |
| 102 | 羅維軍 | 一組家族暴發的2019冠狀病毒病 | 5 | Case report with no additional quantitative data on renal manifestations under investigation |
| 103 | 羅蒙 | 新型冠狀病毒肺炎患者死亡影響因素分析 | 5 | No data collection on renal manifestations |
| 104 | 聞名 | 2019冠狀病毒病患者合并肝損傷的臨床特點 | 5 | No data collection on renal manifestations |
| 105 | 胡曉燕 | 治療新型冠狀病毒肺炎的中藥聯用常用西藥不良相互作用分析 | 5 | Narrative review with no data collection on renal manifestations |
| 106 | 胡美霖 | 中西醫結合治療重癥新型冠狀病毒肺炎臨床病例1例 | 5 | Case report with no additional quantitative data on renal manifestations under investigation |
| 107 | 艾香英 | 廣州地區128例新型冠狀病毒感染患者的臨床特征及預警指標分析 | 5 | No data collection on renal manifestations |
| 108 | 葛艷玲 | 基于8篇病例系列報告和10篇病例報告的兒童新型冠狀病毒肺炎流行病學、臨床特征和出院結局的系統綜述 | 5 | Systematic review with no data collection on renal manifestations. |
| 109 | 董坤 | 新型冠狀病毒肺炎合并糖尿病酮癥酸中毒的臨床診治 | 5 | No data collection on renal manifestations |
| 110 | 董淑杰 | 新型冠狀病毒感染疫情中ACEI的爭議：下結論還為時尚早 | 5 | Narrative review with no data collection on renal manifestations |
| 111 | 董艷迎 | 新型冠狀病毒肺炎患者實驗室檢測指標與免疫學特征分析 | 5 | No data collection on renal manifestations |
| 112 | 袁娜娜 | D-二聚體、C反應蛋白及白細胞介素-6在新型冠狀病毒肺炎的臨床意義及相關性分析 | 5 | No data collection on renal manifestations |
| 113 | 譚鑫 | 長沙市兒童新型冠狀病毒感染13例臨床特征分析 | 5 | Case report with no data collection on renal manifestations |
| 114 | 賈明 | 新型冠狀病毒肺炎文獻整理及研究概述 | 5 | Narrative review with no data collection on renal manifestations |
| 115 | 賴學莉 | 新型冠狀病毒感染疫情下腹膜透析患者的管理 | 5 | Narrative review with no data collection on renal manifestations |
| 116 | 趙紫楠 | α-干擾素霧化吸入的快速衛生技術評估及其在新型冠狀病毒肺炎中應用的可能與價值 | 5 | Narrative review with no data collection on renal manifestations |
| 117 | 郭兆 | 微滴式數字PCR技術在新型冠狀病毒檢測中的潛在價值 | 5 | Narrative review with no data collection on renal manifestations |
| 118 | 郭寧 | 新型冠狀病毒肺炎患者心肌損傷標志物初步探討 | 5 | No data collection on renal manifestations |
| 119 | 郭斌 | 新型冠狀病毒肺炎患者淋巴細胞及炎性指標的動態分析 | 5 | No data collection on renal manifestations |
| 120 | 郭玉嬌 | 洛匹那韋/利托那韋在治療新型冠狀病毒肺炎中的應用價值 | 5 | Narrative review with no data collection on renal manifestations |
| 121 | 郭鴻 | 新型冠狀病毒肺炎與甲型H1N1流感流行病學與臨床特點的對比分析 | 5 | No data collection on renal manifestations |
| 122 | 鄒義龍 | 新型冠狀病毒肺炎患者病情嚴重程度與臨床特征的關系 | 5 | No data collection on renal manifestations |
| 123 | 鄭小豐 | 克力芝與阿比多爾聯合中醫藥治療對新型冠狀病毒肺炎患者腎臟損傷的影響 | 5 | RCT with no reporting on renal manifestations of outcomes |
| 124 | 鄭楷煉 | 糖皮質激素聯合大劑量免疫球蛋白成功治療重型新型冠狀病毒肺炎1例臨床經驗分析 | 5 | Case report with no additional quantitative data on renal manifestations under investigation |
| 125 | 錢志成 | 重型及危重型新型冠狀病毒肺炎的流行病學和臨床特征 | 5 | No data collection on renal manifestations |
| 126 | 陳友惠 | 昆明地區新型冠狀病毒肺炎的臨床特征分析 | 5 | No data collection on renal manifestations |
| 127 | 陳文 | 荊門市第一人民醫院91新型冠狀病毒肺炎患者的臨床特征分析 | 5 | No data collection on renal manifestations |
| 128 | 陳晴 | 成都市新型冠狀病毒肺炎一代病例與二代病例臨床特征分析 | 5 | No data collection on renal manifestations |
| 129 | 陳赟 and 林鳳平 | 糖尿病患者易感新型冠狀病毒肺炎相關性分析 | 5 | No data collection on renal manifestations |
| 130 | 陳香美 | 中國中西醫結合學會腎臟疾病專業委員會致全國中西醫結合腎內科醫師在新型冠狀病毒感染背景下疫情防控和腎臟疾病診治中的防護指導意見 | 5 | Narrative review with no data collection on renal manifestations |
| 131 | 陶飛 | 新型冠狀病毒肺炎患者382例早期臨床特征分析 | 5 | No data collection on renal manifestations |
| 132 | 陸云飛 | 50例新型冠狀病毒感染的肺炎患者中醫臨床特征分析 | 5 | Retrospective cohort with no data collection on renal manifestations |
| 134 | 韓冰 | 感染人類的7種冠狀病毒所致疾病臨床綜述 | 5 | Narrative review with no data collection on renal manifestations |
| 135 | 韓園園 | 中西醫結合治療新型冠狀病毒肺炎的應用分析 | 5 | Narrative review with no data collection on renal manifestations |
| 136 | 馬耀玲 | 115例新型冠狀病毒感染兒童的臨床特點分析 | 5 | Narrative review with no data collection on renal manifestations |
| 137 | 馬耀玲 | 115例新型冠狀病毒感染兒童的臨床特點分析 | 5 | No data collection on renal manifestations |
| 138 | 馬青龍 | 新型冠狀病毒細胞受體ACE2的研究進展 | 5 | Narrative review with no data collection on renal manifestations |
| 139 | 馮歡歡 | 新型冠狀病毒感染臨床分型的探討 | 5 | No data collection on renal manifestations |
| 140 | 馮淬靈 | 2019冠狀病毒病124例患者舌象分析 | 5 | No data collection on renal manifestations |
| 141 | 高思哲 | 新型冠狀病毒肺炎的心血管系統表現 | 5 | No data collection on renal manifestations |
| 142 | 魏方軍 | 新型冠狀病毒肺炎不同臨床分型影像特征的對比性分析 | 5 | No data collection on renal manifestations |
| 143 | 黃亞雄 | 新型冠狀病毒肺炎出院病例121例分析 | 5 | No data collection on renal manifestations |
| 144 | 黃仙保 | 熱敏灸治療新型冠狀病毒肺炎臨床觀察 | 5 | No data collection on renal manifestations |
| 145 | 黃子通 | 2019冠狀病毒病（COVID-19）與嚴重急性呼吸綜合征（SARS） | 5 | Narrative review with no data collection on renal manifestations |
| 146 | 黃春明 | 103例伴消化道癥狀新型冠狀病毒肺炎患者的臨床特征 | 5 | No data collection on renal manifestations |
| 147 | 黃春明 | 新型冠狀病毒肺炎合并肝臟生化學異常的特點 | 5 | No data collection on renal manifestations |
| 148 | 黃煒 | 老年人新型冠狀病毒肺炎死亡病例1例分析 | 5 | Case report with no additional quantitative data on renal manifestations under investigation |
| 149 | 龔雪 | 225例新型冠狀病毒肺炎的臨床特征及中醫藥應用分析 | 5 | No data collection on renal manifestations |
| 150 | Struyf, T | Signs and symptoms to determine if a patient presenting in primary care or hospital outpatient settings has COVID?19 disease | 5 | Systematic review |
| 151 | Abdelmotagly, Y | Investigating the rationale for extending ct kub to include the chest for suspected renal colic patients during the covid-19 pandemic | 5 | No data collection on renal manifestations |
| 152 | Abrishami, M | Ocular Manifestations of Hospitalized Patients with COVID-19 in Northeast of Iran | 5 | No data collection on renal manifestations |
| 154 | Al-Samkari, H | COVID and Coagulation: bleeding and Thrombotic Manifestations of SARS-CoV2 Infection | 5 | No data collection on renal manifestations |
| 155 | Amat-Santos, I | Ramipril in High Risk Patients with COVID-19 | 5 | No data collection on renal manifestations |
| 156 | Asgharpour, M | Effectiveness of extracorporeal blood purification (hemoadsorption) in patients with severe coronavirus disease 2019 (COVID-19) | 5 | No data collection on renal manifestations |
| 157 | Atal, S | IL-6 Inhibitors in the Treatment of Serious COVID-19: a Promising Therapy? | 5 | No data collection on renal manifestations |
| 158 | Ayerbe, L | The association between treatment with heparin and survival in patients with Covid-19 | 5 | No data collection on renal manifestations |
| 159 | B, C | QT prolongation in a diverse, urban population of COVID-19 patients treated with hydroxychloroquine, chloroquine, or azithromycin | 5 | No data collection on renal manifestations |
| 160 | Beigel, J | Remdesivir for the Treatment of Covid-19 - Preliminary Report | 5 | No data collection on renal manifestations |
| 161 | Calabrese, C | Practical aspects of targeting IL-6 in COVID-19 disease | 5 | No data collection on renal manifestations |
| 162 | Calabrese, L | Cytokine release syndrome and the prospects for immunotherapy with COVID-19. Part 2: the role of interleukin 1 | 5 | No data collection on renal manifestations |
| 163 | Carignan, A | Anosmia and dysgeusia associated with SARS-CoV-2 infection: an age-matched case-control study | 5 | No data collection on renal manifestations |
| 164 | Castelnuovo, A | Use of hydroxychloroquine in hospitalised COVID-19 patients is associated with reduced mortality: findings from the observational multicentre Italian CORIST study | 5 | No data collection on renal manifestations |
| 165 | Cavalcanti, A | Hydroxychloroquine with or without Azithromycin in Mild-to-Moderate Covid-19 | 3 | Included suspected COVID-19 |
| 166 | Chatterjee, K | Steroids in COVID-19: an overview | 5 | No data collection on renal manifestations |
| 167 | Chen, B | Early experience with convalescent plasma as immunotherapy for COVID-19 in China: knowns and unknowns | 5 | No data collection on renal manifestations |
| 168 | Chevalier, M | Hydroxychloroquine/ chloroquine as a treatment choice or prophylaxis for Covid-19 at the primary care level in developing countries: a Primum non Nocere dilemma | 5 | No data collection on renal manifestations |
| 169 | Chiaravalloti, N | The emotional impact of the COVID-19 pandemic on individuals with progressive multiple sclerosis | 5 | No data collection on renal manifestations |
| 170 | Cilia, R | Effects of COVID-19 on Parkinson's Disease Clinical Features: a Community-Based Case-Control Study | 5 | No data collection on renal manifestations |
| 171 | Dai, M | Patients with Cancer Appear More Vulnerable to SARS-CoV-2: a Multicenter Study during the COVID-19 Outbreak | 5 | No data collection on renal manifestations |
| 172 | de Chaisemartin, C | BCG vaccination in infancy does not protect against COVID-19. Evidence from a natural experiment in Sweden | 5 | No data collection on renal manifestations |
| 173 | Deana, C | The COVID-19 pandemic: is our medicine still evidence-based? | 5 | No data collection on renal manifestations |
| 174 | Del Amo, J | Incidence and Severity of COVID-19 in HIV-Positive Persons Receiving Antiretroviral Therapy: a Cohort Study | 5 | No data collection on renal manifestations |
| 175 | Doggrell, S | Does lopinavir measure up in the treatment of COVID-19? | 5 | No data collection on renal manifestations |
| 176 | Farkash, E | Ultrastructural evidence for direct renal infection with sars-cov-2 | 5 | No data collection on renal manifestations |
| 177 | Frohman, E | Part I. SARS-CoV-2 triggered ‘PANIC’ attack in severe COVID-19 | 5 | No data collection on renal manifestations |
| 178 | Frohman, E | Part II. high-dose methotrexate with leucovorin rescue for severe COVID-19: an immune stabilization strategy for SARS-CoV-2 induced ‘PANIC’1 attack | 5 | No data collection on renal manifestations |
| 179 | Fürstenau, M | COVID-19 among fit patients with CLL treated with venetoclax-based combinations | 5 | No data collection on renal manifestations |
| 180 | Goldman, J | Remdesivir for 5 or 10 Days in Patients with Severe Covid-19 | 5 | Randomised controlled trial with unclear definition of AKI |
| 181 | Gong, Y | Effects of methylprednisolone use on viral genomic nucleic acid negative conversion and CT imaging lesion absorption in COVID-19 patients under 50 years old | 5 | No data collection on renal manifestations |
| 182 | Horby, P | Dexamethasone in Hospitalized Patients with Covid-19 - Preliminary Report | 5 | No data collection on renal manifestations |
| 183 | Huang, Y | No Statistically Apparent Difference in Antiviral Effectiveness Observed Among Ribavirin Plus Interferon-Alpha, Lopinavir/Ritonavir Plus Interferon-Alpha, and Ribavirin Plus Lopinavir/Ritonavir Plus Interferon-Alpha in Patients With Mild to Moderate Coronavirus Disease 2019: results of a Randomized, Open-Labeled Prospective Study | 5 | No data collection on renal manifestations |
| 184 | Jalessi, M | Frequency and outcome of olfactory impairment and sinonasal involvement in hospitalized patients with COVID-19 | 5 | No data collection on renal manifestations |
| 185 | Jendrny, P | Scent dog identification of samples from COVID-19 patients - a pilot study | 5 | No data collection on renal manifestations |
| 186 | Keller, M | Effect of systemic glucocorticoids on mortality or mechanical ventilation in patients with COVID-19 | 5 | No data collection on renal manifestations |
| 187 | Khamis, F | Therapeutic Plasma Exchange in Adults with Severe COVID-19 Infection | 5 | No data collection on renal manifestations |
| 188 | Khan, Z | Anti COVID-19 Drugs: need for More Clinical Evidence and Global Action | 5 | No data collection on renal manifestations |
| 189 | Kim, J | Lopinavir-ritonavir versus hydroxychloroquine for viral clearance and clinical improvement in patients with mild to moderate coronavirus disease 2019 | 5 | No data collection on renal manifestations |
| 190 | Klok, F | Incidence of thrombotic complications in critically ill ICU patients with COVID-19 | 5 | No data collection on renal manifestations |
| 192 | Li, J | Application of CareDose 4D combined with Karl 3D technology in the low dose computed tomography for the follow-up of COVID-19 | 5 | No data collection on renal manifestations |
| 193 | Li, L | Retrospective Study of Risk Factors for Myocardial Damage in Patients With Critical Coronavirus Disease 2019 in Wuhan | 5 | No data collection on renal manifestations |
| 194 | Li, Z | From community-acquired pneumonia to COVID-19: a deep learning–based method for quantitative analysis of COVID-19 on thick-section CT scans | 5 | No data collection on renal manifestations |
| 195 | Lian, N | Umifenovir treatment is not associated with improved outcomes in patients with coronavirus disease 2019: a retrospective study | 5 | No data collection on renal manifestations |
| 196 | Littlejohn, E | Hydroxychloroquine use in the COVID-19 patient | 5 | No data collection on renal manifestations |
| 197 | Liu, K | Respiratory rehabilitation in elderly patients with COVID-19: a randomized controlled study | 5 | No data collection on renal manifestations |
| 198 | Liu, K | Effects of progressive muscle relaxation on anxiety and sleep quality in patients with COVID-19 | 5 | No data collection on renal manifestations |
| 199 | Liu, X | Efficacy of chloroquine versus lopinavir/ritonavir in mild/general COVID-19 infection: a prospective, open-label, multicenter, randomized controlled clinical study | 5 | No data collection on renal manifestations |
| 200 | Maziarz, M | Agent-based modelling for SARS-CoV-2 epidemic prediction and intervention assessment: a methodological appraisal | 5 | No data collection on renal manifestations |
| 201 | Meletiadis, J | Interleukin-6 Blocking vs. JAK-STAT Inhibition for Prevention of Lung Injury in Patients with COVID-19 | 5 | No data collection on renal manifestations |
| 202 | Mulligan, M | Phase 1/2 study of COVID-19 RNA vaccine BNT162b1 in adults | 5 | No data collection on renal manifestations |
| 203 | Nelson, B | Clinical Outcomes Associated with Methylprednisolone in Mechanically Ventilated Patients with COVID-19 | 5 | No data collection on renal manifestations |
| 204 | Nili, A | Remdesivir: a beacon of hope from Ebola virus disease to COVID-19 | 5 | No data collection on renal manifestations |
| 205 | Olender, S | Remdesivir for Severe COVID-19 versus a Cohort Receiving Standard of Care | 5 | No data collection on renal manifestations |
| 206 | Ong, S | Safety and potential efficacy of cyclooxygenase-2 inhibitors in coronavirus disease 2019 | 5 | No data collection on renal manifestations |
| 207 | Ou-Yang, J | Blood Donor Recruitment in Guangzhou, China, during the 2019 Novel Coronavirus (COVID-19) Epidemic | 5 | No data collection on renal manifestations |
| 208 | Patell, R | Post-discharge thrombosis and hemorrhage in patients with COVID-19 | 5 | No data collection on renal manifestations |
| 210 | Plaze, M | Repurposing chlorpromazine to treat COVID-19: the reCoVery study | 5 | No data collection on renal manifestations |
| 211 | Powell-Jackson, T | Infection prevention and control compliance in Tanzanian outpatient facilities: a cross-sectional study with implications for the control of COVID-19 | 5 | No data collection on renal manifestations |
| 212 | Price, C | Tocilizumab treatment for Cytokine Release Syndrome in hospitalized COVID-19 patients: survival and clinical outcomes | 5 | No data collection on renal manifestations |
| 213 | Quartuccio, L | Profiling COVID-19 pneumonia progressing into the cytokine storm syndrome: results from a single Italian Centre study on tocilizumab versus standard of care | 5 | No data collection on renal manifestations |
| 214 | Ramiro, S | Historically controlled comparison of glucocorticoids with or without tocilizumab versus supportive care only in patients with COVID-19-associated cytokine storm syndrome: results of the CHIC study | 5 | No data collection on renal manifestations |
| 215 | Rao, S | Exploring Diseases/Traits and Blood Proteins Causally Related to Expression of ACE2, the Putative Receptor of SARS-CoV-2: a Mendelian Randomization Analysis Highlights Tentative Relevance of Diabetes-Related Traits | 5 | No data collection on renal manifestations |
| 216 | Ratnasekera, N | Supportive care for oral cancer survivors in COVID-19 lockdown | 5 | No data collection on renal manifestations |
| 217 | Rayman, G | Dexamethasone therapy in COVID-19 patients: implications and guidance for the management of blood glucose in people with and without diabetes | 5 | No data collection on renal manifestations |
| 218 | Rieder, M | Cytokine adsorption in patients with severe COVID-19 pneumonia requiring extracorporeal membrane oxygenation | 5 | No data collection on renal manifestations |
| 219 | Rivera-Izquierdo, M | Therapeutic agents tested in 238 COVID-19 hospitalized patients and their relationship with mortality | 5 | No data collection on renal manifestations |
| 220 | Roberts, L | Post-discharge venous thromboembolism following hospital admission with COVID-19 | 5 | No data collection on renal manifestations |
| 221 | Rothlin, R | Telmisartan as tentative angiotensin receptor blocker therapeutic for COVID-19 | 5 | No data collection on renal manifestations |
| 222 | Sahajpal, N | Proposal of Reverse Transcription-PCR-Based Mass Population Screening for SARS-CoV-2 (COVID-19) | 5 | No data collection on renal manifestations |
| 223 | Schwartz, R | Azithromycin and COVID-19Prompt Early Use at First Signs of this Infection in Adults and Children An Approach Worthy of Consideration | 5 | No data collection on renal manifestations |
| 224 | Sheng, C | Canakinumab to reduce deterioration of cardiac and respiratory function in SARS-CoV-2 associated myocardial injury with heightened inflammation (canakinumab in Covid-19 cardiac injury: the three C study) | 5 | No data collection on renal manifestations |
| 226 | Shu, L | Treatment of severe COVID-19 with human umbilical cord mesenchymal stem cells | 5 | No data collection on renal manifestations |
| 227 | Sibley, C | Effects of the COVID-19 pandemic and nationwide lockdown on trust, attitudes toward government, and well-being | 5 | No data collection on renal manifestations |
| 228 | Singh, A | A pharmacovigilance study of hydroxychloroquine cardiac safety profile: potential implication in COVID‐19 mitigation | 5 | No data collection on renal manifestations |
| 229 | Slim, K | The wave of “opinion articles” in the coverage of COVID-19 in surgical literature | 5 | No data collection on renal manifestations |
| 230 | Son, W | Individual-based simulation model for COVID-19 transmission in Daegu,Korea | 5 | No data collection on renal manifestations |
| 231 | Stringhini, S | Seroprevalence of anti-SARS-CoV-2 IgG antibodies in Geneva, Switzerland (SEROCoV-POP): a population-based study | 5 | No data collection on renal manifestations |
| 232 | Sverzellati, N | Integrated Radiologic Algorithm for COVID-19 Pandemic | 5 | No data collection on renal manifestations |
| 233 | Ul Alam, Asmr, Rafiul Islam, M | Understanding the possible origin and genotyping of first Bangladeshi SARS-CoV-2 strain | 5 | No data collection on renal manifestations |
| 234 | Vetrovsky, T | The detrimental effect of COVID-19 nationwide quarantine on accelerometer-assessed physical activity of heart failure patients | 5 | No data collection on renal manifestations |
| 235 | Viecca, M | Enhanced platelet inhibition treatment improves hypoxemia in patients with severe Covid-19 and hypercoagulability. A case control, proof of concept study | 5 | No data collection on renal manifestations |
| 236 | Waller, J | 'Immunity Passports' for SARS-CoV-2: an online experimental study of the impact of antibody test terminology on perceived risk and behaviour | 5 | No data collection on renal manifestations |
| 237 | Wang, Y | The Risk of Children Hospitalized With Severe COVID-19 in Wuhan | 5 | No data collection on renal manifestations |
| 238 | Wei, N | Efficacy of internet-based integrated intervention on depression and anxiety symptoms in patients with COVID-19 | 5 | No data collection on renal manifestations |
| 239 | Wen, L | Effect of Xuebijing injection on inflammatory markers and disease outcome of coronavirus disease 2019 | 5 | No data collection on renal manifestations |
| 240 | Wong, Ay- Y | Impact of the COVID-19 pandemic on sports and exercise | 5 | No data collection on renal manifestations |
| 241 | Wu, S | Identification and validation of a novel clinical signature to predict the prognosis in confirmed COVID-19 patients | 5 | No data collection on renal manifestations |
| 242 | Xu, P | Arbidol/IFN-α2b therapy for patients with corona virus disease 2019: a retrospective multicenter cohort study | 5 | No data collection on renal manifestations |
| 243 | Yan, B | Large- scale prospective clinical study on prophylactic intervention of COVID-19 in community population using Huoxiang Zhengqi Oral Liquid and Jinhao Jiere Granules | 5 | Randomised controlled trial with no data collection on renal manifestations |
| 244 | Yang, G | Effects Of ARBs And ACEIs On Virus Infection, Inflammatory Status And Clinical Outcomes In COVID-19 Patients With Hypertension: a Single Center Retrospective Study | 5 | No data collection on renal manifestations |
| 245 | Ye, Y | Guideline-Based Chinese Herbal Medicine Treatment Plus Standard Care for Severe Coronavirus Disease 2019 (G-CHAMPS): evidence From China | 5 | Randomised controlled trial with no data collection on renal manifestations |
| 246 | Yuan, N | Investigation of adverse reactions in healthcare personnel working in Level 3 barrier protection PPE to treat COVID-19 | 5 | No data collection on renal manifestations |
| 247 | Yufei, Y | Utility of the neutrophil-to-lymphocyte ratio and C-reactive protein level for coronavirus disease 2019 (COVID-19) | 5 | No data collection on renal manifestations |
| 248 | Zhang, X | In-Hospital Use of Statins Is Associated with a Reduced Risk of Mortality among Individuals with COVID-19 | 5 | No data on renal manifestations of interest |
| 249 | Zhao, J | Yidu-toxicity blocking lung decoction ameliorates inflammation in severe pneumonia of SARS-COV-2 patients with Yidu-toxicity blocking lung syndrome by eliminating IL-6 and TNF-a | 5 | No data on renal manifestations of interest |
| 250 | Afewerky, H | Pathology and pathogenicity of severe acute respiratory syndrome coronavirus 2 (SARS-CoV-2) | 5 | No data collection on renal manifestations |
| 252 | Alberici, F. | Management of Patients on Dialysis and With Kidney Transplantation During the SARS-CoV-2 (COVID-19) Pandemic in Brescia, Italy | 5 | Narrative review |
| 253 | Al-Jameel, W | Similarities and differences of covid-19 and avian infectious bronchitis from molecular pathologist and poultry specialist view point | 5 | No data collection on renal manifestations |
| 254 | Antonio, R. | Immunosuppression drug-related and clinical manifestation of Coronavirus disease 2019: a therapeutical hypothesis | 5 | Narrative review |
| 255 | Arenas, M | Protection of nephrology health professionals during the COVID-19 pandemic | 5 | No data collection on renal manifestations |
| 256 | Auld, S | ICU and Ventilator Mortality among Critically Ill Adults with Coronavirus Disease 2019* | 5 | No data collection on renal manifestations |
| 257 | Bartiromo, M. | Threatening drug-drug interaction in a kidney transplant patient with Coronavirus Disease 2019 (COVID-19) | 5 | Case report with no additional quantitative data on renal manifestations under investigation |
| 258 | Bello-Chavolla, O | Predicting Mortality Due to SARS-CoV-2: A Mechanistic Score Relating Obesity and Diabetes to COVID-19 Outcomes in Mexico | 5 | No data collection on renal manifestations |
| 259 | Bhandari, S | Characteristics, Treatment Outcomes and Role of Hydroxychloroquine among 522 COVID-19 hospitalized patients in Jaipur City: An Epidemio-Clinical Study | 1 | Full text not retrievable |
| 260 | Bulut, C | Epidemiology of covid-19 | 5 | No data collection on renal manifestations |
| 261 | Bussalino, E. | Immunosuppressive therapy maintenance in a kidney transplant recipient SARS-CoV-2 pneumonia: a case report | 5 | Case report with no additional quantitative data on renal manifestations under investigation |
| 262 | Cappell, M | Moderately Severe Diarrhea and Impaired Renal Function With COVID-19 Infection | 5 | No data collection on renal manifestations |
| 263 | Chen, S. | Clinical analysis of pregnant women with 2019 novel coronavirus pneumonia | 5 | No data collection on renal manifestations |
| 265 | Chen, T | Clinical Characteristics and Outcomes of Older Patients with Coronavirus Disease 2019 (COVID-19) in Wuhan, China: A Single-Centered, Retrospective Study | 5 | No data collection on renal manifestations |
| 266 | Cheng, H. | Organ-protective effect of angiotensin-converting enzyme 2 and its effect on the prognosis of COVID-19 | 5 | Narrative review |
| 268 | Chilimuri, S | Predictors of Mortality in Adults Admitted with COVID-19: Retrospective Cohort Study from New York City | 5 | No data collection on renal manifestations |
| 269 | de Souza Silva, G | SARS-CoV, MERS-CoV and SARS-CoV-2 infections in pregnancy and fetal development | 5 | No data collection on renal manifestations |
| 270 | Deng, Y. | Clinical characteristics of fatal and recovered cases of coronavirus disease 2019 (COVID-19) in Wuhan, China: a retrospective study | 5 | Case control study. Labeled as retrospective cohort. Location and period overlap with #8 and #57 with no additional quantitative data on renal manifestations under investigation |
| 271 | Escher, R | Severe COVID-19 infection associated with endothelial activation | 5 | No data collection on renal manifestations |
| 272 | Fang, L. | Are patients with hypertension and diabetes mellitus at increased risk for COVID-19 infection? | 5 | Narrative review |
| 273 | Faqihi, F | Reverse takotsubo cardiomyopathy in fulminant COVID-19 associated with cytokine release syndrome and resolution following therapeutic plasma exchange: a case-report | 5 | No data collection on renal manifestations |
| 274 | Fara, A | Cytokine storm and COVID-19: a chronicle of pro-inflammatory cytokines | 5 | No data collection on renal manifestations |
| 275 | Ferrey, A. J. | A Case of Novel Coronavirus Disease 19 in a Chronic Hemodialysis Patient Presenting with Gastroenteritis and Developing Severe Pulmonary Disease | 5 | Case report with no data collection on renal manifestations |
| 276 | Fox, S | Unexpected Features of Cardiac Pathology in COVID-19 Infection | 5 | No data collection on renal manifestations |
| 277 | Fried, J | The Variety of Cardiovascular Presentations of COVID-19 | 5 | No data collection on renal manifestations |
| 278 | Fu, D. | COVID-19 Infection in a Patient with End-Stage Kidney Disease | 5 | Case report with no data collection on renal manifestations |
| 280 | Gedney, N | Long-term hemodialysis during the COVID-19 pandemic | 5 | No data collection on renal manifestations |
| 281 | Gregoriano, C | Characteristics, predictors and outcomes among 99 patients hospitalised with COVID-19 in a tertiary care centre in Switzerland: an observational analysis | 5 | No data collection on renal manifestations |
| 282 | Gu, M. | Analysis of property and efficacy of traditional Chinese medicine in staging revention and treatment of coronavirus disease 2019. [Chinese] | 5 | Data mining on treatment utilisation with no clinical data |
| 283 | Guillen, E. | Case report of COVID-19 in a kidney transplant recipient: Does immunosuppression alter the clinical presentation? | 5 | Case report with no data collection on renal manifestations |
| 284 | Harrison, S | Comorbidities associated with mortality in 31,461 adults with COVID-19 in the United States: A federated electronic medical record analysis | 5 | No data collection on renal manifestations |
| 285 | Hay, R | A viral rash: the impact of COVID-19 infection on the skin | 5 | No data collection on renal manifestations |
| 287 | Henry, B. M. | Hematologic, biochemical and immune biomarker abnormalities associated with severe illness and mortality in coronavirus disease 2019 (COVID-19): a meta-analysis | 5 | Systematic review with no data collection on renal manifestations |
| 288 | Henry, B. M. | Chronic kidney disease is associated with severe coronavirus disease 2019 (COVID-19) infection | 5 | Systematic review with no data collection on renal manifestations |
| 289 | Huang, X | Bioinformatic Analysis of Correlation between Immune Infiltration and COVID-19 in Cancer Patients | 5 | No data collection on renal manifestations |
| 290 | Hultstrom, M | Hyperreninemia and low total body water may contribute to acute kidney injury in COVID-19 patients in intensive care | 5 | No data collection on renal manifestations |
| 292 | Hussain, A. | COVID-19 and diabetes: Knowledge in progress | 5 | Narrative review with no data collection on renal manifestations |
| 293 | Iaccarino, G | Age and Multimorbidity Predict Death among COVID-19 Patients: Results of the SARS-RAS Study of the Italian Society of Hypertension | 5 | No data collection on renal manifestations |
| 294 | Jamous, F | Critical Illness Due to Covid-19: A Description of the Surge in a Single Center in Sioux Falls | 1 | Full text not retrievable |
| 295 | Jazieh, A | Outcome of oncology patients infected with coronavirus | 3 | Not COVID related on renal manifestations |
| 296 | Ji, W | Effect of Underlying Comorbidities on the Infection and Severity of COVID-19 in Korea: a Nationwide Case-Control Study | 5 | No data collection on renal manifestations |
| 297 | Jin, M | Rhabdomyolysis as Potential Late Complication Associated with COVID-19 | 5 | No data collection on renal manifestations |
| 298 | Johnson, K | Pulmonary and Extra-Pulmonary Clinical Manifestations of COVID-19 | 5 | No data collection on renal manifestations |
| 299 | Kammar-Garcia, A | Impact of Comorbidities in Mexican Sars-Cov-2-Positive Patients: A Retrospective Analysis in a National Cohort | 5 | No data collection on renal manifestations |
| 300 | Karami, P. | Mortality of a pregnant patient diagnosed with COVID-19: A case report with clinical, radiological, and histopathological findings | 5 | Case report with no data collection on renal manifestations |
| 301 | Keller, N | Impact of first-wave COronaVIrus disease 2019 infection in patients on haemoDIALysis in Alsace: the observational COVIDIAL study | 5 | No data collection on renal manifestations |
| 302 | Killerby, M | Characteristics Associated with Hospitalization Among Patients with COVID-19 - Metropolitan Atlanta, Georgia, March-April 2020 | 5 | No data collection on renal manifestations |
| 303 | Kim, D | The Correlation of Comorbidities on the Mortality in Patients with COVID-19: an Observational Study Based on the Korean National Health Insurance Big Data | 5 | No data collection on renal manifestations |
| 304 | Kreutz, R. | Hypertension, the renin-angiotensin system, and` the risk of lower respiratory tract infections and lung injury: implications for COVID-19 | 5 | Narrative review with no data collection on renal manifestations |
| 305 | Lai, C. C. | Severe acute respiratory syndrome coronavirus 2 (SARS-CoV-2) and coronavirus disease-2019 (COVID-19): The epidemic and the challenges | 5 | Narrative review with no data collection on renal manifestations |
| 306 | Larsen, C. P. | Collapsing Glomerulopathy in a Patient With Coronavirus Disease 2019 (COVID-19) | 5 | Case report |
| 307 | Le, M | Pharmacokinetics of lopinavir/ritonavir oral solution to treat COVID-19 in mechanically ventilated ICU patients | 5 | No data collection on renal manifestations |
| 308 | Lee, J | Kidney transplantation trends in South Korea during the COVID-19 pandemic | 5 | No data collection on renal manifestations |
| 309 | Li, H | Biochemical analysis between common type and critical type of COVID-19 and clinical value of neutrophil/lymphocyte ratio. [Chinese] | 5 | No data collection on renal manifestations |
| 310 | Li, H | Extra-pulmonary clinical manifestations of novel coronavirus pneumonia. [Chinese] | 5 | No data collection on renal manifestations |
| 311 | Lillie, P. J. | Novel coronavirus disease (Covid-19): The first two patients in the UK with person to person transmission | 5 | Case report with no data collection on renal manifestations |
| 313 | Little, P. | Non-steroidal anti-inflammatory drugs and covid-19 | 5 | Narrative review with no data collection on renal manifestations |
| 314 | Liu, J | Application of continuous renal replacement therapy in coronavirus disease 2019. [Chinese] | 5 | No data collection on renal manifestations |
| 315 | Liu, L | Clinical characteristics of hospitalized patients with 2019 novel coronavirus disease indicate potential proximal tubular dysfunction | 5 | No data collection on renal manifestations |
| 316 | Liu, W | Clinical Analysis of Neonates Born to Mothers with or without COVID-19: A Retrospective Analysis of 48 Cases from Two Neonatal Intensive Care Units in Hubei Province | 5 | No data collection on renal manifestations |
| 318 | Lopez, V. | Recommendations on management of the SARS-CoV-2 coronavirus pandemic (Covid-19) in kidney transplant patients | 5 | Narrative review with no data collection on renal manifestations |
| 321 | Marx, D. | First case of COVID-19 in a kidney transplant recipient treated with belatacept | 5 | Case report with no data collection on renal manifestations |
| 322 | Meijers, B | The clinical characteristics of coronavirus-associated nephropathy | 5 | No data collection on renal manifestations |
| 323 | Miller, R | Clinical presentation and course of COVID-19 | 5 | No data collection on renal manifestations |
| 324 | Mubarak, M. | Covid-19 nephropathy; an emerging condition caused by novel coronavirus infection | 5 | Editorial with no data collection on renal manifestations |
| 326 | Naicker, S | The Novel Coronavirus 2019 epidemic and kidneys | 5 | No data collection on renal manifestations |
| 327 | Naneishvili, T | Fulminant myocarditis as an early presentation of SARS-CoV-2 | 5 | No data collection on renal manifestations |
| 328 | Ng, L. F. P. | Coronaviruses in animals and humans | 5 | Narrative review with no data collection on renal manifestations |
| 329 | Oberweis, M | Pediatric Life-Threatening Coronavirus Disease 2019 With Myocarditis | 5 | No data collection on renal manifestations |
| 330 | Pang, W. T. | Analysis on pattern of prescriptions and syndromes of traditional Chinese medicine for prevention and treatment of COVID-19. [Chinese] | 5 | Narrative review with no data collection on renal manifestations |
| 331 | Peng, Y | Clinical characteristics and outcomes of 112 cardiovascular disease patients infected by 2019-nCoV. [Chinese] | 5 | No data collection on renal manifestations |
| 332 | Ponce Diaz-Reixa, J | Renal transplantation during COVID-19 period in Spain. [Spanish] | 1 | Irretrievable |
| 334 | Puelles, V | Multiorgan and Renal Tropism of SARS-CoV-2 | 5 | No data collection on renal manifestations |
| 335 | Qi, F. | Single cell RNA sequencing of 13 human tissues identify cell types and receptors of human coronaviruses | 4 | In silico |
| 336 | Radmanesh, A | COVID-19-associated delayed posthypoxic necrotizing leukoencephalopathy | 5 | No data collection on renal manifestations |
| 337 | Rasmussen, S. A. | Coronavirus Disease 2019 (COVID-19) and pregnancy: what obstetricians need to know | 5 | Narrative review with no data collection on renal manifestations |
| 338 | Razzaghi, H | Estimated County-Level Prevalence of Selected Underlying Medical Conditions Associated with Increased Risk for Severe COVID-19 Illness - United States, 2018 | 5 | No data collection on renal manifestations |
| 339 | Rezende, L | Adults at high-risk of severe coronavirus disease-2019 (Covid-19) in Brazil | 5 | No data collection on renal manifestations |
| 340 | Rismanbaf, A | Liver and kidney injuries in COVID-19 and their effects on drug therapy; a letter to editor | 5 | No data collection on renal manifestations |
| 341 | Roncati, L. | Renin-angiotensin system: The unexpected flaw inside the human immune system revealed by SARS-CoV-2 | 5 | Narrative review with no data collection on renal manifestations |
| 342 | Ronco, C. | Coronavirus epidemic: preparing for extracorporeal organ support in intensive care | 5 | Narrative review with no data collection on renal manifestations |
| 343 | Rossi, G. P. | Potential harmful effects of discontinuing ACE-inhibitors and ARBs in COVID-19 patients | 5 | Narrative review with no data collection on renal manifestations |
| 344 | Saavedra, J. M. | Angiotensin receptor blockers and COVID-19 | 5 | Narrative review with no data collection on renal manifestations |
| 345 | Samies, N | Rhabdomyolysis and Acute Renal Failure in an Adolescent With Coronavirus Disease 2019 | 5 | No data collection on renal manifestations |
| 346 | Santos, M | Survival and predictors of deaths of patients hospitalised due to COVID-19 from a retrospective and multicentre cohort study in Brazil | 5 | No data collection on renal manifestations |
| 347 | Schweitzer, W. | Implications for forensic death investigations from first Swiss post-mortem CT in a case of non-hospital treatment with COVID-19 | 8 | Forensic investigation |
| 348 | Senemaud, J | Intraoperative adverse events and early outcomes of custom-made fenestrated stent grafts and physician-modified stent grafts for complex aortic aneurysms | 5 | No data collection on renal manifestations |
| 350 | Singhal, T. | A Review of Coronavirus Disease-2019 (COVID-19) | 5 | Narrative review with no data collection on renal manifestations |
| 352 | Su, H. | Renal histopathological analysis of 26 postmortem findings of patients with COVID-19 in China | 5 | Autopsy reports with no additional quantitative data on renal manifestations under investigation |
| 353 | Tian, S. | Pathological study of the 2019 novel coronavirus disease (COVID-19) through postmortem core biopsies | 8 | Biopsy report with no data on renal manifestations |
| 355 | Wang, L. | Coronavirus Disease 19 Infection Does Not Result in Acute Kidney Injury: An Analysis of 116 Hospitalized Patients from Wuhan, China | 5 | Retrospective cohort. Location and period overlap with #349 |
| 356 | Wang, T | Comorbidities and multi-organ injuries in the treatment of COVID-19 | 5 | No data collection on renal manifestations |
| 357 | Wichmann, D | Autopsy Findings and Venous Thromboembolism in Patients With COVID-19: A Prospective Cohort Study | 5 | No data collection on renal manifestations |
| 358 | Xiao, Y. | Severe Acute Respiratory Syndrome Coronavirus 2 Infection in Renal Failure Patients: A Potential Covert Source of Infection | 5 | Narrative review with no data collection on renal manifestations |
| 359 | Xu, H | Clinical Characteristics and Risk Factors of Cardiac Involvement in COVID-19 | 5 | No data collection on renal manifestations |
| 360 | Yang, F | Analysis of 92 deceased patients with COVID-19 | 5 | No data collection on renal manifestations |
| 362 | Yang, Z. | Predictors for imaging progression on chest CT from coronavirus disease 2019 (COVID-19) patients | 5 | Retrospective cohort with no quantitative data of renal manifestations |
| 363 | Zhan, W. Q. | Successful treatment of COVID-19 using extracorporeal membrane oxygenation, a case report | 5 | Case report with no data collection on renal manifestations |
| 364 | Zhang, F. | Potential risk of the kidney vulnerable to novel coronavirus 2019 infection | 5 | Narrative review with no data collection on renal manifestations |
| 365 | Zhang, H. | Identification of Kidney Transplant Recipients with Coronavirus Disease 2019 | 5 | Case report with no quantitative data collection on renal manifestations |
| 367 | Zhao, Z | Interleukin-6 and severity of COVID-19 patients in Hefei, China | 5 | No data collection on renal manifestations |
| 368 | Zhong, Z. | Clinical characteristics and immunosuppressants management of coronavirus disease 2019 in solid organ transplant recipients | 5 | Case report with no data collection on renal manifestations |
| 369 | Zhou, J | Epidemiological and clinical features of 201 COVID-19 patients in Changsha city, Hunan, China | 5 | No data collection on renal manifestations |
| 370 | Zhou, X | Follow-up of asymptomatic patients with SARS-CoV-2 infection | 5 | No data collection on renal manifestations |
| 371 | Zhu, J | Clinicopathological characteristics of 8697 patients with COVID-19 in China: a meta-analysis | 5 | No data collection on renal manifestations |
| 372 | Zhu, J. | Clinical characteristics of 3,062 COVID-19 patients: a meta-analysis | 5 | Meta-analysis |
| 374 | Zhu, L. | Successful recovery of COVID-19 pneumonia in a renal transplant recipient with long-term immunosuppression | 5 | Case report |
| 375 | Abreu, A | The Brazilian Society of Nephrology and the Covid-19 Pandemic | 5 | No data collection on renal manifestations |
| 376 | Abrishami, A | Clinical and Radiologic Characteristics of COVID-19 in Patients With CKD | 5 | No data collection on renal manifestations |
| 377 | Abrishami, A | Clinical Course, Imaging Features, and Outcomes of COVID-19 in Kidney Transplant Recipients | 5 | No data collection on renal manifestations |
| 378 | Adapa, S | COVID-19 in Renal Transplant Patient Presenting With Active Typical Symptoms and Resolved Atypical Symptoms | 5 | Case report |
| 380 | Ahmed, I | Severe pre-eclampsia complicated by acute fatty liver disease of pregnancy, HELLP syndrome and acute kidney injury following SARS-CoV-2 infection | 5 | No data collection on renal manifestations |
| 381 | Ahmed, R | COVID-19 and Impending Shortage of Haemodialysis Facilities due to the Rising Incidence of Acute Kidney Injury Requiring Renal Replacement Therapy | 5 | No data collection on renal manifestations |
| 383 | Akdur, A | Coronavirus Disease (COVID-19) in Kidney and Liver Transplant Patients: A Single-Center Experience | 5 | No data collection on renal manifestations |
| 386 | Arenas, M | [Management of the SARS-CoV-2 (COVID-19) coronavirus epidemic in hemodialysis units] | 5 | No data collection on renal manifestations |
| 389 | Banerjee, D | COVID-19 infection in kidney transplant recipients | 5 | Case report |
| 390 | Barros Camargo, L | Acute kidney injury associated with COVID-19: another extrapulmonary manifestation | 5 | No data collection on renal manifestations |
| 391 | Bhatla, A | COVID-19 and cardiac arrhythmias | 5 | No data collection on renal manifestations |
| 392 | Blumfield, E | COVID-19 in pediatric patients: a case series from the Bronx, NY | 5 | No data collection on renal manifestations |
| 393 | Braun, F | SARS-CoV-2 renal tropism associates with acute kidney injury | 5 | No data collection on demographics |
| 394 | Bravi, F | Predictors of severe or lethal COVID-19, including Angiotensin Converting Enzyme inhibitors and Angiotensin II Receptor Blockers, in a sample of infected Italian citizens | 5 | No data collection on renal manifestations |
| 395 | Cao, J | Myocardial injury and COVID-19: Serum hs-cTnI level in risk stratification and the prediction of 30-day fatality in COVID-19 patients with no prior cardiovascular disease | 5 | No data collection on renal manifestations |
| 396 | Casey, K | COVID-19 pneumonia with hemoptysis: Acute segmental pulmonary emboli associated with novel coronavirus infection | 5 | No data collection on renal manifestations |
| 397 | Cavalcanti, D | Cerebral Venous Thrombosis Associated with COVID-19 | 5 | No data collection on renal manifestations |
| 399 | Chapman, A | High-Sensitivity Cardiac Troponin Can Be an Ally in the Fight Against COVID-19 | 5 | No data collection on demographics |
| 400 | Chen, D | Assessment of Hypokalemia and Clinical Characteristics in Patients With Coronavirus Disease 2019 in Wenzhou, China | 5 | No data collection on renal manifestations |
| 401 | Chen, L | Elevated serum levels of S100A8/A9 and HMGB1 at hospital admission are correlated with inferior clinical outcomes in COVID-19 patients | 5 | No data collection on renal manifestations |
| 402 | Chen, S | A familial cluster, including a kidney transplant recipient, of Coronavirus Disease 2019 (COVID-19) in Wuhan, China | 5 | No data collection on renal manifestations |
| 403 | Chen, T. | Clinical characteristics of 113 deceased patients with coronavirus disease 2019: retrospective study | 6 | Duplicated with #264 |
| 405 | Cho, J | Hemodialysis with Cohort Isolation to Prevent Secondary Transmission during a COVID-19 Outbreak in Korea | 5 | No data collection on renal manifestations |
| 406 | Ciceri, F | Early predictors of clinical outcomes of COVID-19 outbreak in Milan, Italy | 5 | No data collection on renal manifestations |
| 407 | Corbett, R | Epidemiology of COVID-19 in an Urban Dialysis Center | 5 | No data collection on renal manifestations |
| 408 | Cui, X | Acute Kidney Injury in Patients with the Coronavirus Disease 2019: A Multicenter Study | 5 | No data collection on overall demographics |
| 409 | Cumhur Cure, M | NSAIDs may increase the risk of thrombosis and acute renal failure in patients with COVID-19 infection | 5 | No data collection on overall demographics |
| 410 | Cummings, M | Epidemiology, clinical course, and outcomes of critically ill adults with COVID-19 in New York City: a prospective cohort study | 5 | Overlapped with #387 |
| 411 | Darbani, B | The Expression and Polymorphism of Entry Machinery for COVID-19 in Human: Juxtaposing Population Groups, Gender, and Different Tissues | 5 | No data collection on overall demographics |
| 412 | de Lusignan, S | Risk factors for SARS-CoV-2 among patients in the Oxford Royal College of General Practitioners Research and Surveillance Centre primary care network: a cross-sectional study | 5 | No data collection on overall demographics |
| 413 | Deep, A | Acute Kidney Injury and COVID-19: Attention to Inflammatory Phenotype | 5 | No data collection on overall demographics |
| 415 | Fanelli, V. | Acute kidney injury in SARS-CoV-2 infected patients | 5 | Narrative review with no data collection on renal manifestations |
| 417 | Forouzesh, M | Clinical display, diagnostics and genetic implication of novel Coronavirus (COVID-19) epidemic | 5 | No data collection on renal manifestations |
| 418 | Frydman, G | Coagulation Status and Venous Thromboembolism Risk in African Americans: A Potential Risk Factor in COVID-19 | 5 | No data collection on renal manifestations |
| 419 | Fu, D | COVID-19 Infection in a Patient with End-Stage Kidney Disease | 5 | No data collection on renal manifestations |
| 420 | Gabarre, P | Acute kidney injury in critically ill patients with COVID-19 | 5 | No data collection on renal manifestations |
| 421 | Gandolfini, I | COVID-19 in kidney transplant recipients | 5 | No data collection on renal manifestations |
| 422 | Gao, Q | The epidemiological characteristics of 2019 novel coronavirus diseases (COVID-19) in Jingmen, Hubei, China | 5 | No data collection on renal manifestations |
| 423 | Gisondi, P | Risk of hospitalization and death from COVID-19 infection in patients with chronic plaque psoriasis receiving a biologic treatment and renal transplant recipients in maintenance immunosuppressive treatment | 5 | No data collection on renal manifestations |
| 424 | Goldfarb, D | Impending Shortages of Kidney Replacement Therapy for COVID-19 Patients | 5 | No data collection on renal manifestations |
| 425 | Gu, M. | [Analysis of property and efficacy of traditional Chinese medicine in staging revention and treatment of coronavirus disease 2019] | 6 | Duplicated with #282 |
| 426 | Gupta, A | Extrapulmonary manifestations of COVID-19 | 5 | No data collection on renal manifestations |
| 427 | Haberal, M. | Covid-19 Update | 5 | Editorial with no data collection on renal manifestations |
| 428 | Helms, J | High risk of thrombosis in patients with severe SARS-CoV-2 infection: a multicenter prospective cohort study | 5 | No data collection on renal manifestations |
| 429 | Henry, B | Hematologic, biochemical and immune biomarker abnormalities associated with severe illness and mortality in coronavirus disease 2019 (COVID-19): a meta-analysis | 5 | No data collection on renal manifestations |
| 430 | Henry, B | Chronic kidney disease is associated with severe coronavirus disease 2019 (COVID-19) infection | 5 | No data collection on renal manifestations |
| 431 | Hernandez-Garduno, E | Obesity is the comorbidity more strongly associated for Covid-19 in Mexico. A case-control study | 5 | No data collection on renal manifestations |
| 433 | Ho, Q | High-immunological risk living donor renal transplant during the COVID-19 outbreak: Uncertainties and ethical dilemmas | 5 | No data collection on renal manifestations |
| 435 | Hsu, J | COVID-19 in a high-risk dual heart and kidney transplant recipient | 5 | No data collection on renal manifestations |
| 436 | Huang, W | [Effects of angiotensin converting enzyme inhibitor/angiotensin receptor blocker on clinical characteristics of coronavirus disease 2019 patients with hypertension] | 5 | No data collection on renal manifestations |
| 437 | Husain, S | Early Outcomes of Outpatient Management of Kidney Transplant Recipients with Coronavirus Disease 2019 | 5 | No data collection on renal manifestations |
| 438 | Hwang, J | Neurological diseases as mortality predictive factors for patients with COVID-19: a retrospective cohort study | 5 | No data collection on renal manifestations |
| 439 | Iaccarino, G | Age and Multimorbidity Predict Death Among COVID-19 Patients: Results of the SARS-RAS Study of the Italian Society of Hypertension | 5 | No data collection on renal manifestations |
| 440 | Ikizler, T | Minimizing the risk of COVID-19 among patients on dialysis | 5 | No data collection on renal manifestations |
| 441 | Ippolito, M | [Standard procedures in dialysis during the Covid-19 epidemic] | 5 | No data collection on renal manifestations |
| 442 | Ji, H. L. | Elevated Plasmin(ogen) as a Common Risk Factor for COVID-19 Susceptibility | 5 | Narrative review with no data collection on renal manifestations |
| 443 | Jiang, S | [Clinical feature changes of a COVID-19 patient from mild to critical condition and cardiopulmonary pathological results] | 5 | No data collection on renal manifestations |
| 444 | John, J | Imminent risk of COVID-19 in diabetes mellitus and undiagnosed diabetes mellitus patients | 5 | No data collection on renal manifestations |
| 445 | Joyner, M | Early safety indicators of COVID-19 convalescent plasma in 5000 patients | 5 | No data collection on renal manifestations |
| 446 | Kadosh, B | Collapsing glomerulopathy associated with COVID-19 infection in a heart transplant recipient | 5 | No data collection on renal manifestations |
| 447 | Kajani, R | Neuroleptic malignant syndrome in a COVID-19 patient | 5 | No data collection on renal manifestations |
| 449 | Khamis, F | Clinical characteristics and outcomes of the first 63 adult patients hospitalized with COVID-19: An experience from Oman | 5 | No data collection on renal manifestations |
| 450 | Khurshid, Z. | Human Saliva: Non-Invasive Fluid for Detecting Novel Coronavirus (2019-nCoV) | 5 | Narrative review with no data collection on renal manifestations |
| 451 | Kliger, A | Mitigating Risk of COVID-19 in Dialysis Facilities | 5 | No data collection on renal manifestations |
| 452 | Kudose, S | Kidney Biopsy Findings in Patients with COVID-19 | 5 | No data collection on renal manifestations |
| 453 | La Vignera, S | Sex-Specific SARS-CoV-2 Mortality: Among Hormone-Modulated ACE2 Expression, Risk of Venous Thromboembolism and Hypovitaminosis D | 5 | No data collection on renal manifestations |
| 454 | Lai, C | Extra-respiratory manifestations of COVID-19 | 5 | No data collection on renal manifestations |
| 455 | Lauretani, F | Assessment and treatment of older individuals with COVID 19 multi-system disease: Clinical and ethical implications | 5 | No data collection on renal manifestations |
| 457 | Lerner, A | A kidney transplant recipient with nausea, vomiting, and diarrhea after a recent COVID-19 exposure | 5 | No data collection on renal manifestations |
| 458 | Li, D | Immune dysfunction leads to mortality and organ injury in patients with COVID-19 in China: insights from ERS-COVID-19 study | 5 | No data collection on renal manifestations |
| 459 | Li, H | [Extra-pulmonary clinical manifestations of novel coronavirus pneumonia] | 5 | No data collection on renal manifestations |
| 460 | Li, H | [Biochemical analysis between common type and critical type of COVID-19 and clinical value of neutrophil/lymphocyte ratio] | 5 | No data collection on renal manifestations |
| 461 | Liu, C | [Preliminary study of the relationship between novel coronavirus pneumonia and liver function damage: a multicenter study] | 5 | No data collection on renal manifestations |
| 462 | Liu, J | [Application of continuous renal replacement therapy in coronavirus disease 2019] | 5 | No data collection on renal manifestations |
| 463 | Liu, X | Analysis of clinical features and early warning signs in patients with severe COVID-19: A retrospective cohort study | 5 | No data collection on renal manifestations |
| 464 | Liu, Y | [Clinical analysis of kidney injury in patients with COVID-19] | 6 | Duplicated with #317 |
| 465 | Lu, J | [Clinical characteristics and outcomes of adult critically ill patients with COVID-19 in Honghu, Hubei Province] | 6 | Duplicated with #319 |
| 466 | Lun, Y | [Comparative analysis of structural characteristics and epitopes in S proteins between SARS-CoV-2 and SARS-CoV] | 5 | No data collection on renal manifestations |
| 467 | Lushina, N | Pulmonary, Cerebral, and Renal Thromboembolic Disease in a Patient with COVID-19 | 5 | No data collection on renal manifestations |
| 468 | Man, Z | Viral shedding prolongation in a kidney transplant patient with COVID-19 pneumonia | 5 | No data collection on renal manifestations |
| 469 | Manes, M | [Preliminary report on the Covid-19 outbreak in Valle d'Aosta dialysis centers] | 5 | No data collection on renal manifestations |
| 470 | Manna, S | Spontaneous subcutaneous emphysema and pneumomediastinum in non-intubated patients with COVID-19 | 5 | No data collection on renal manifestations |
| 471 | Marlais, M | The severity of COVID-19 in children on immunosuppressive medication | 5 | No data collection on renal manifestations |
| 472 | Martino, F | Kidney transplant programmes during the COVID-19 pandemic | 5 | No data collection on renal manifestations |
| 474 | Merhi, B | Kidney Transplantation and COVID-19 | 1 | Full text not retrievable |
| 475 | Mihalopoulos, M | COVID-19 and Kidney Disease: Molecular Determinants and Clinical Implications in Renal Cancer | 5 | No data collection on renal manifestations |
| 476 | Mo, J | Predictive role of clinical features in patients with coronavirus disease 2019 for severe disease | 5 | No data collection on renal manifestations |
| 477 | Mohamed, M | Renal Transplant Recipient with Concurrent COVID-19 and Stenotrophomonas maltophilia Pneumonia Treated with Trimethoprim/Sulfamethoxazole Leading to Acute Kidney Injury: A Therapeutic Dilemma | 5 | No data collection on renal manifestations |
| 478 | Nagatomo, M | Peritoneal dialysis for COVID-19-associated acute kidney injury | 5 | No data collection on renal manifestations |
| 481 | Nalleballe, K | Spectrum of neuropsychiatric manifestations in COVID-19 | 5 | No data collection on renal manifestations |
| 482 | Namazee, N | Novel coronavirus 2019 pneumonia in a kidney transplant recipient | 5 | No data collection on renal manifestations |
| 483 | Nikpouraghdam, M | Epidemiological characteristics of coronavirus disease 2019 (COVID-19) patients in IRAN: A single center study | 5 | No data collection on renal manifestations |
| 484 | Niquini, R | Description and comparison of demographic characteristics and comorbidities in SARI from COVID-19, SARI from influenza, and the Brazilian general population | 5 | No data collection on renal manifestations |
| 485 | Okoh, A | Coronavirus disease 19 in minority populations of Newark, New Jersey | 5 | No data collection on renal manifestations |
| 487 | Ozturk, R | COVID-19: pathogenesis, genetic polymorphism, clinical features and laboratory findings | 5 | No data collection on renal manifestations |
| 488 | Pachiega, J | Chronic heart diseases as the most prevalent comorbidities among deaths by COVID-19 in Brazil | 5 | No data collection on renal manifestations |
| 490 | Pan, P | [The timing of continuous renal replacement therapy in severe COVID-19] | 5 | No data collection on renal manifestations |
| 492 | Pang, W. T. | [Analysis on pattern of prescriptions and syndromes of traditional Chinese medicine for prevention and treatment of COVID-19] | 6 | Duplicated with #330 |
| 493 | Pascual Gomez, N | [Potential biomarkers predictors of mortality in COVID-19 patients in the Emergency Department] | 5 | No data collection on renal manifestations |
| 494 | Pascual, J | COVID-19-related Mortality During the First 60 Days After Kidney Transplantation | 5 | No data collection on renal manifestations |
| 495 | Patel, M | Retrospective analysis of high flow nasal therapy in COVID-19-related moderate-to-severe hypoxaemic respiratory failure | 5 | No data collection on renal manifestations |
| 498 | Peng, Y | [Clinical characteristics and outcomes of 112 cardiovascular disease patients infected by 2019-nCoV] | 6 | Duplicated with #331 |
| 499 | Pereira, A | Clinical course of coronavirus disease-2019 in pregnancy | 5 | No data collection on renal manifestations |
| 500 | Pereira, M | Severe clinical spectrum with high mortality in pediatric patients with COVID-19 and multisystem inflammatory syndrome | 5 | No data collection on renal manifestations |
| 501 | Pereira, M | COVID-19 in solid organ transplant recipients: Initial report from the US epicenter | 5 | No data collection on renal manifestations |
| 502 | Pesaresi, M | SARS-CoV-2 identification in lungs, heart and kidney specimens by transmission and scanning electron microscopy | 5 | No data collection on renal manifestations |
| 503 | Petrilli, C | Factors associated with hospital admission and critical illness among 5279 people with coronavirus disease 2019 in New York City: prospective cohort study | 5 | No data collection on renal manifestations |
| 504 | Post, A | Kidney Infarction in Patients With COVID-19 | 5 | No data collection on renal manifestations |
| 506 | Qu, J | Clinical characteristics of COVID-19 and its comparison with influenza pneumonia | 5 | No data collection on renal manifestations |
| 507 | Ren, Z | Epidemiologic and clinical characteristics of heart transplant recipients during the 2019 coronavirus outbreak in Wuhan, China: A descriptive survey report | 5 | No data collection on renal manifestations |
| 509 | Sachdeva, M | COVID-19 in Hospitalized Patients on Chronic Peritoneal Dialysis: A Case Series | 5 | No data collection on renal manifestations |
| 510 | Samavat, S | COVID-19 Rapid Guideline in Kidney Transplant Recipients | 5 | No data collection on renal manifestations |
| 511 | Sanchez-Alvarez, J | [SARS-CoV-2 infection in patients on renal replacement therapy. Report of the COVID-19 Registry of the Spanish Society of Nephrology (SEN)] | 5 | No data collection on renal manifestations |
| 512 | Santoriello, D | Postmortem Kidney Pathology Findings in Patients with COVID-19 | 5 | No data collection on renal manifestations |
| 513 | Shi, S | Characteristics and clinical significance of myocardial injury in patients with severe coronavirus disease 2019 | 5 | No data collection on renal manifestations |
| 514 | Soares, R | Risk Factors for Hospitalization and Mortality due to COVID-19 in Espirito Santo State, Brazil | 5 | No data collection on renal manifestations |
| 516 | Soliman, A | Prevalence, clinical manifestations, and biochemical data of type 2 diabetes mellitus versus nondiabetic symptomatic patients with COVID-19: A comparative study | 5 | No data collection on renal manifestations |
| 517 | Sousa, G | Mortality and survival of COVID-19 | 5 | No data collection on renal manifestations |
| 518 | South, A. M. | COVID-19, ACE2, and the cardiovascular consequences | 5 | Narrative review with no data collection on renal manifestations |
| 519 | Stachel, M | COVID-19 pneumonia in a dual heart-kidney recipient | 5 | No data collection on renal manifestations |
| 520 | Stewart, D | Renal dysfunction in hospitalised children with COVID-19 | 5 | No data collection on renal manifestations |
| 521 | Su, Y | Cardiovascular manifestation and treatment in COVID-19 | 5 | No data collection on renal manifestations |
| 523 | Sun, C | [Clinical analysis of 150 cases of 2019 novel coronavirus infection in Nanyang City, Henan Province] | 5 | No data collection on renal manifestations |
| 525 | Szabados, B | Clinical Characteristics and Outcome for Four SARS-CoV-2-infected Cancer Patients Treated with Immune Checkpoint Inhibitors | 5 | No data collection on renal manifestations |
| 526 | Tabatabai, A | Factor VIII and Functional Protein C Activity in Critically Ill Patients With Coronavirus Disease 2019: A Case Series | 5 | No data collection on renal manifestations |
| 527 | Taxbro, K | Rhabdomyolysis and acute kidney injury in severe COVID-19 infection | 5 | No data collection on renal manifestations |
| 528 | Thomas, T | COVID-19 infection alters kynurenine and fatty acid metabolism, correlating with IL-6 levels and renal status | 5 | No data collection on renal manifestations |
| 530 | Tram, N | Rhabdomyolysis and Acute Kidney Injury as Leading COVID-19 Presentation in an Adolescent | 5 | No data collection on renal manifestations |
| 531 | Tuma, J | [Renal Monomorphology in COVID-19 with Acute Renal Insufficiency] | 5 | No data collection on renal manifestations |
| 532 | Turcotte, J | Risk factors for severe illness in hospitalized Covid-19 patients at a regional hospital | 5 | No data collection on renal manifestations |
| 533 | Uribarri, A | Impact of renal function on admission in COVID-19 patients: an analysis of the international HOPE COVID-19 (Health Outcome Predictive Evaluation for COVID 19) Registry | 5 | No data collection on renal manifestations |
| 535 | Vega-Vega, O | [Prevention and control of SARS-CoV-2 (Covid-19) coronavirus infection in hemodialysis units.] | 5 | No data collection on renal manifestations |
| 536 | Vila-Corcoles, A | [Evaluation of incidence and risk profile for suffering Covid-19 infection by underlying conditions among middle-aged and older adults in Tarragona.] | 5 | No data collection on renal manifestations |
| 537 | Wang, B. | Does comorbidity increase the risk of patients with COVID-19: evidence from meta-analysis | 5 | Systematic review with no data collection on renal manifestations |
| 538 | Wang, D | Clinical course and outcome of 107 patients infected with the novel coronavirus, SARS-CoV-2, discharged from two hospitals in Wuhan, China | 5 | Overlap with #225 |
| 539 | Wang, L | Coronavirus Disease 19 Infection Does Not Result in Acute Kidney Injury: An Analysis of 116 Hospitalized Patients from Wuhan, China | 6 | Overlap with #355 |
| 540 | Wang, T | Clinical Features of Coronavirus Disease 2019 Patients With Mechanical Ventilation: A Nationwide Study in China | 5 | No data collection on renal manifestations |
| 541 | Wang, Y | Clinical characteristics of patients with uremia undergoing maintenance hemodialysis complicated with COVID-19 | 5 | No data collection on renal manifestations |
| 542 | Wang, Y | Strategies to halt 2019 novel coronavirus (SARS-CoV-2) spread for organ transplantation programs at the Sichuan Academy of Medical Science and Sichuan Provincial People's Hospital, China | 5 | No data collection on renal manifestations |
| 543 | Wei, J | Acute myocardial injury is common in patients with COVID-19 and impairs their prognosis | 5 | No data collection on renal manifestations |
| 544 | Wei, X | Characteristics of in peripheral blood of 70 hospitalized patients and 8 diarrhea patients with COVID-19 | 5 | No data collection on renal manifestations |
| 545 | Wu, H | AKI and Collapsing Glomerulopathy Associated with COVID-19 and APOL 1 High-Risk Genotype | 5 | No data collection on renal manifestations |
| 546 | Wu, J | Clinical Features of Maintenance Hemodialysis Patients with 2019 Novel Coronavirus-Infected Pneumonia in Wuhan, China | 5 | No data collection on renal manifestations |
| 547 | Wu, Y | [Clinical features and outcome of treatment for novel coronavirus pneumonia: a meta-analysis] | 5 | No data collection on renal manifestations |
| 549 | Xia, T | Coronavirus disease 2019 and transplantation: The combination of lopinavir/ritonavir and hydroxychloroquine is responsible for excessive tacrolimus trough level and unfavorable outcome | 5 | No data collection on renal manifestations |
| 550 | Xiao, Y | Severe Acute Respiratory Syndrome Coronavirus 2 Infection in Renal Failure Patients: A Potential Covert Source of Infection | 5 | No data collection on renal manifestations |
| 551 | Xiong, F | Clinical Characteristics of and Medical Interventions for COVID-19 in Hemodialysis Patients in Wuhan, China | 5 | No data collection on renal manifestations |
| 553 | Xu, K | Application of ordinal logistic regression analysis to identify the determinants of illness severity of COVID-19 in China | 5 | No data collection on renal manifestations |
| 554 | Xu, M | [Clinical analysis of 23 patients with coronavirus disease 2019 in Xinyang City of Henan Province] | 5 | No data collection on renal manifestations |
| 555 | Yamada, T | Patients with chronic kidney disease have a poorer prognosis of coronavirus disease 2019 (COVID-19): an experience in New York City | 5 | No data collection on renal manifestations |
| 556 | Yan, Y | Clinical characteristics and outcomes of patients with severe covid-19 with diabetes | 5 | No data collection on renal manifestations |
| 557 | Yang, C | Hemodialysis vascular access care during the COVID-19 pandemic | 5 | No data collection on renal manifestations |
| 558 | Yang, Q | Analysis of the clinical characteristics, drug treatments and prognoses of 136 patients with coronavirus disease 2019 | 5 | No data collection on renal manifestations |
| 559 | Yang, R | The role of essential organ-based comorbidities in the prognosis of COVID-19 infection patients | 5 | No data collection on renal manifestations |
| 560 | Yang, X | Clinical course and outcomes of critically ill patients with SARS-CoV-2 pneumonia in Wuhan, China: a single-centered, retrospective, observational study | 6 | Duplicated with #361 |
| 561 | Yang, X | [Diagnosis and treatment of COVID-19: acute kidney injury cannot be ignored] | 5 | No data collection on renal manifestations |
| 562 | Yao, X | [A pathological report of three COVID-19 cases by minimal invasive autopsies] | 5 | No data collection on renal manifestations |
| 563 | Yu, C | Epidemiological and clinical characteristics of 1663 hospitalized patients infected with COVID-19 in Wuhan, China: a single-center experience | 5 | No data collection on renal manifestations |
| 564 | Zaim, S | COVID-19 and Multiorgan Response | 5 | No data collection on renal manifestations |
| 565 | Zhang, H | Identification of Kidney Transplant Recipients with Coronavirus Disease 2019 | 5 | No data collection on renal manifestations |
| 566 | Zhang, H | Solid Organ Transplantation During the COVID-19 Pandemic | 5 | Narrative review |
| 567 | Zhang, J | [Clinical characteristics and risk factors of acute kidney injury in coronavirus disease 2019] | 5 | Overlap with #465 |
| 568 | Zhao, J | Risk factors for the exacerbation of patients with 2019 Novel Coronavirus: A meta-analysis | 5 | No data collection on renal manifestations |
| 569 | Zhao, M | Comparison of clinical characteristics and outcomes of patients with coronavirus disease 2019 at different ages | 6 | Duplicated with #366 |
| 570 | Zhao, R | COVID-19 Outbreak and Management Approach for Families with Children on Long-Term Kidney Replacement Therapy | 5 | No data collection on renal manifestations |
| 571 | Zhao, X | Clinical characteristics of patients with 2019 coronavirus disease in a non-Wuhan area of Hubei Province, China: a retrospective study | 5 | No data collection on renal manifestations |
| 573 | Zhong, Z | Clinical characteristics and immunosuppressant management of coronavirus disease 2019 in solid organ transplant recipients | 5 | No data collection on renal manifestations |
| 574 | Zhu, L | Coronavirus Disease 2019 Pneumonia in Immunosuppressed Renal Transplant Recipients: A Summary of 10 Confirmed Cases in Wuhan, China | 5 | No data collection on renal manifestations |
| 575 | Zhu, L | Successful recovery of COVID-19 pneumonia in a renal transplant recipient with long-term immunosuppression | 5 | No data collection on renal manifestations |
| 576 | Zhu, Y | Evaluation of organ function in patients with severe COVID-19 infections | 5 | No data collection on renal manifestations |
| 578 | Zolk, O | COVID-19 pandemic and therapy with ibuprofen or renin-angiotensin system blockers: no need for interruptions or changes in ongoing chronic treatments | 5 | No data collection on renal manifestations |
| 579 | Zou, R | Clinical characteristics and outcome of hemodialysis patients with COVID-19: a large cohort study in a single Chinese center | 5 | No data collection on renal manifestations |
| 582 | Alberici, F | A report from the Brescia Renal COVID Task Force on the clinical characteristics and short-term outcome of hemodialysis patients with SARS-CoV-2 infection | 5 | No data collection on renal manifestations |
| 583 | Al-Samkari, H | COVID-19 and coagulation: bleeding and thrombotic manifestations of SARS-CoV-2 infection | 5 | No data collection on renal manifestations |
| 584 | Auld, S | ICU and Ventilator Mortality Among Critically Ill Adults With Coronavirus Disease 2019 | 5 | No data collection on renal manifestations |
| 585 | Azoulay, E | Increased mortality in patients with severe SARS-CoV-2 infection admitted within seven days of disease onset | 6 | Duplicated with #388 |
| 586 | Batlle, D. | Soluble angiotensin-converting enzyme 2: a potential approach for coronavirus infection therapy? | 5 | Narrative review with no data collection on renal manifestations |
| 587 | Bello-Chavolla, O | Predicting Mortality Due to SARS-CoV-2: A Mechanistic Score Relating Obesity and Diabetes to COVID-19 Outcomes in Mexico | 5 | No data collection on renal manifestations |
| 588 | Bruminhent, J | Clinical characteristics and risk factors for coronavirus disease 2019 (COVID-19) among patients under investigation in Thailand | 5 | No data collection on renal manifestations |
| 589 | Canevelli, M | COVID-19 mortality among migrants living in Italy | 5 | No data collection on renal manifestations |
| 590 | Cheng, A | Diagnostic performance of initial blood urea nitrogen combined with D-dimer levels for predicting in-hospital mortality in COVID-19 patients | 5 | No data collection on renal manifestations |
| 591 | Corbett, R | Epidemiology of COVID-19 in an Urban Dialysis Center | 5 | No data collection on renal manifestations |
| 592 | De Smet, R | Frailty and Mortality in Hospitalized Older Adults With COVID-19: Retrospective Observational Study | 5 | No data collection on renal manifestations |
| 593 | Deng, M | Obesity as a Potential Predictor of Disease Severity in Young COVID-19 Patients: A Retrospective Study | 5 | No data collection on renal manifestations |
| 594 | Deshpande, R | Study of COVID-19 Pandemic in Representative Dialysis Population Across Mumbai, India: An Observational Multicentric Analysis | 1 | Irretrievable |
| 595 | Dirim, A | Fatal SARS-CoV-2 infection in a renal transplant recipient | 5 | No data collection on renal manifestations |
| 596 | Docherty, A | Features of 20 133 UK patients in hospital with covid-19 using the ISARIC WHO Clinical Characterisation Protocol: prospective observational cohort study | 5 | No data collection on renal manifestations |
| 597 | Dong, X | Prognostic value of lactate dehydrogenase for in-hospital mortality in severe and critically ill patients with COVID-19 | 5 | No data collection on renal manifestations |
| 598 | Emmi, G | SARS-CoV-2 infection among patients with systemic autoimmune diseases | 5 | No data collection on renal manifestations |
| 599 | Feng, J | Safety and treatment completion of latent tuberculosis infection treatment in the elderly population-A prospective observational study in Taiwan | 5 | No data collection on renal manifestations |
| 600 | Fernández-Ruiz, M | COVID-19 in solid organ transplant recipients: A single-center case series from Spain | 5 | No data collection on renal manifestations |
| 601 | Goicoechea, M | COVID-19: clinical course and outcomes of 36 hemodialysis patients in Spain | 5 | No data collection on renal manifestations |
| 602 | Han, H | Profiling serum cytokines in COVID-19 patients reveals IL-6 and IL-10 are disease severity predictors | 5 | No data collection on renal manifestations |
| 603 | He, Y | Clinical Characteristics and Outcomes of Patients with Severe COVID-19 and Chronic Obstructive Pulmonary Disease (COPD) | 5 | No data collection on renal manifestations |
| 604 | Helms, J | High risk of thrombosis in patients with severe SARS-CoV-2 infection: a multicenter prospective cohort study | 6 | Duplicated with #428 |
| 605 | Hirsch, J | Acute kidney injury in patients hospitalized with COVID-19 | 6 | Duplicated with #432 |
| 606 | Holman, N | Risk factors for COVID-19-related mortality in people with type 1 and type 2 diabetes in England: a population-based cohort study | 5 | No data collection on renal manifestations |
| 607 | Iaccarino, G. | Renin-Angiotensin System Inhibition in Cardiovascular Patients at the Time of COVID19: Much Ado for Nothing? A Statement of Activity from the Directors of the Board and the Scientific Directors of the Italian Society of Hypertension | 5 | Narrative review with no data collection on renal manifestations |
| 609 | Karagiannidis, C | Case characteristics, resource use, and outcomes of 10 021 patients with COVID-19 admitted to 920 German hospitals: an observational study | 6 | Duplicated with #448 |
| 610 | Kikuchi, K | COVID-19 of dialysis patients in Japan: Current status and guidance on preventive measures | 5 | No data collection on renal manifestations |
| 611 | Korth, J | SARS-CoV-2-specific antibody detection in healthcare workers in Germany with direct contact to COVID-19 patients | 5 | No data collection on renal manifestations |
| 613 | Lendorf, M | Characteristics and early outcomes of patients hospitalised for COVID-19 in North Zealand, Denmark | 6 | Duplicated with #456 |
| 614 | Lippi, G | Electrolyte imbalances in patients with severe coronavirus disease 2019 (COVID-19) | 5 | No data collection on renal manifestations |
| 615 | Liu, B | Epidemiological characteristics of COVID-19 patients in convalescence period | 5 | No data collection on renal manifestations |
| 616 | Liu, J | Critically ill patients with COVID-19 with ECMO and artificial liver plasma exchange: A retrospective study | 5 | No data collection on renal manifestations |
| 617 | Lund, L | Adverse outcomes and mortality in users of non-steroidal anti-inflammatory drugs who tested positive for SARS-CoV-2: A Danish nationwide cohort study | 5 | No data collection on renal manifestations |
| 618 | Mahévas, M | Clinical efficacy of hydroxychloroquine in patients with covid-19 pneumonia who require oxygen: observational comparative study using routine care data | 5 | No data collection on renal manifestations |
| 619 | McCullough, P. A. | Urgent need for individual mobile phone and institutional reporting of at home, hospitalized, and intensive care unit cases of SARS-CoV-2 (COVID-19) infection | 5 | Comments with no data collection on renal manifestations |
| 620 | Mo, J | Predictive role of clinical features in patients with coronavirus disease 2019 for severe disease | 5 | No data collection on renal manifestations |
| 621 | Murillo-Zamora, E | Male gender and kidney illness are associated with an increased risk of severe laboratory-confirmed coronavirus disease | 5 | No data collection on renal manifestations |
| 622 | Ng, B | Lung computed tomography patterns of a cluster of asymptomatic young males with COVID-19 admitted to a teaching hospital in Kuala Lumpur | 5 | No data collection on renal manifestations |
| 623 | Nikoo, M | Electrocardiographic findings of methanol toxicity: a cross-sectional study of 356 cases in Iran | 5 | No data collection on renal manifestations |
| 624 | Ntaios, G | Characteristics and Outcomes in Patients With COVID-19 and Acute Ischemic Stroke: The Global COVID-19 Stroke Registry | 5 | No data collection on renal manifestations |
| 625 | Paital, B | Inter nation social lockdown versus medical care against COVID-19, a mild environmental insight with special reference to India | 5 | No data collection on renal manifestations |
| 627 | Pascual Gómez, N | [Potential biomarkers predictors of mortality in COVID-19 patients in the Emergency Department] | 5 | No data collection on renal manifestations |
| 628 | Portolés, J | Chronic kidney disease and acute kidney injury in the COVID-19 Spanish outbreak | 6 | Duplicated with #333 |
| 629 | Poulton, K | A role for human leucocyte antigens in the susceptibility to SARS-Cov-2 infection observed in transplant patients | 5 | No data collection on renal manifestations |
| 630 | Richardson, S | Presenting Characteristics, Comorbidities, and Outcomes Among 5700 Patients Hospitalized With COVID-19 in the New York City Area | 6 | Duplicated with #508 |
| 631 | Shi, S | Association of Cardiac Injury With Mortality in Hospitalized Patients With COVID-19 in Wuhan, China | 6 | Duplicated with #349 |
| 632 | Soares, R | Risk Factors for Hospitalization and Mortality due to COVID-19 in Espírito Santo State, Brazil | 5 | No data collection on renal manifestations |
| 633 | Sousa, G | Mortality and survival of COVID-19 | 5 | No data collection on renal manifestations |
| 634 | Sud, A | Collateral damage: the impact on outcomes from cancer surgery of the COVID-19 pandemic | 5 | No data collection on renal manifestations |
| 635 | Sun, H | Comparison of clinical and microbiological diagnoses for older adults with COVID-19 in Wuhan: a retrospective study | 5 | No data collection on renal manifestations |
| 636 | Sun, H | Risk Factors for Mortality in 244 Older Adults With COVID-19 in Wuhan, China: A Retrospective Study | 5 | No data collection on renal manifestations |
| 637 | Talreja, H. | A consensus statement on the use of angiotensin receptor blockers and angiotensin converting enzyme inhibitors in relation to COVID-19 (corona virus disease 2019) | 5 | Viewpoint with no data collection on renal manifestations |
| 638 | Uribarri, A | Impact of renal function on admission in COVID-19 patients: an analysis of the international HOPE COVID-19 (Health Outcome Predictive Evaluation for COVID 19) Registry | 6 | Duplicated with #533 |
| 639 | Varim, C | Neutrophil count to albumin ratio as a new predictor of mortality in patients with COVID-19 ınfection | 5 | No data collection on renal manifestations |
| 640 | Vila-Córcoles, Á, Ochoa-Gondar, O | [Evaluation of incidence and risk profile for suffering Covid-19 infection by underlying conditions among middle-aged and older adults in Tarragona.] | 5 | No data collection on renal manifestations |
| 641 | Wan, Y | Prognosis analysis of patients with mental disorders with COVID-19: a single-center retrospective study | 5 | No data collection on renal manifestations |
| 642 | Wang, X | Ratios of neutrophil-to-lymphocyte and platelet-to-lymphocyte predict all-cause mortality in inpatients with coronavirus disease 2019 (COVID-19): a retrospective cohort study in a single medical centre | 5 | No data collection on renal manifestations |
| 643 | Wang, Z | A Retrospective Study from 2 Centers in China on the Effects of Continued Use of Angiotensin-Converting Enzyme Inhibitors and Angiotensin II Receptor Blockers in Patients with Hypertension and COVID-19 | 5 | No data collection on renal manifestations |
| 644 | Wichmann, D | Autopsy Findings and Venous Thromboembolism in Patients With COVID-19: A Prospective Cohort Study | 6 | Duplicated with #357 |
| 645 | Wu, Y | Relationship between ABO blood group distribution and clinical characteristics in patients with COVID-19 | 5 | No data collection on renal manifestations |
| 646 | Wynants, L. | Prediction models for diagnosis and prognosis of covid-19 infection: systematic review and critical appraisal | 5 | Systematic review on prediction models with no quantitative data on renal manifestations |
| 647 | Xu, X | Seroprevalence of immunoglobulin M and G antibodies against SARS-CoV-2 in China | 5 | No data collection on renal manifestations |
| 648 | Zhang, M. Q. | [Clinical features of 2019 novel coronavirus pneumonia in the early stage from a fever clinic in Beijing] | 5 | Retrospective cohort with no data collection on renal manifestations |
| 649 | 刘洋 | 新型冠状病毒肺炎患者肾损伤的临床分析 | 6 | Duplicated with #317 |
| 650 | 刘熙 | 火神山医院新型冠状病毒肺炎47例死亡病例流行病学特点及临床特征分析 | 5 | No data collection on renal manifestations |
| 651 | 劉澗 | 新型冠状病毒肺炎(普通型)与疑似患者临床特征比较 | 6 | Duplicated with #18 |
| 652 | 张丽慧 | 成都市新型冠状病毒肺炎患者的临床特征分析 | 6 | Duplicated with #54 |
| 653 | 张嘉皓 | 新型冠状病毒肺炎相关性急性肾损伤的临床特点及危险因素分析 | 6 | Duplicated with #567 |
| 654 | 杨继 | 2 831例新型冠状病毒肺炎患者中医证素特点的文献调查分析 | 5 | No data collection on renal manifestations |
| 655 | 林柳 | 新型冠状病毒肺炎患者并发急性肾损伤的临床分析 | 6 | Duplicated with #78 |
| 656 | 薛红 | 新型冠状病毒感染第一代、第二代患者临床特征分析 | 5 | No data collection on renal manifestations |
| 657 | 陶飞 | 新型冠状病毒肺炎患者382例早期临床特征分析 | 5 | No data collection on renal manifestations |
| 658 |  | 中国杭州市重症监护室34例COVID-19患者临床特点的分析 | 1 | Full text not retrievable |

^ 1. Full-text irretrievable, 2. Language untranslatable, 3. Not COVID-related, 4. Non-human study, 5. No original quantitative data of interest, 6. Duplicate**.**

**S3 Characteristics of included studies for meta-analysis**

| **First Author** | **Title** | **Country** | **City** | **Hospital** | **Sampling period** | **Sampling population** | **Sample size** | **Consecutive or random sampling** | **Study design** |
| --- | --- | --- | --- | --- | --- | --- | --- | --- | --- |
| Cheng, Y. | Kidney disease is associated with in-hospital death of patients with COVID-19 | China | Wuhan | Tongji Hospital | 28 January 2020 to 11 February 2020 | General population with COVID19. Pediatric patients and patients with a history of maintenance dialysis or renal transplantation were excluded | 701 | Yes | Prospective cohort |
| Ling, L. | Critically ill patients with COVID-19 in Hong Kong: a multicentre retrospective observational cohort study | China | Hong Kong | Prince of Wales Hospital, Princess Margaret Hospital, Pamela Youde Nethersole Eastern Hospital | 22 January 2020 to 11 February 2020 | All adult critically ill patients with confirmed COVID19 admitted to ICU | 8 | Yes | Retrospective cohort |
| Shi, S. | Association of Cardiac Injury with Mortality in Hospitalized Patients with COVID-19 in Wuhan, China | China | Wuhan | Renmin Hospital | 20 January 2020 to 10 February 2020 | General population with COVID19 with cardiac biomarker investigations. | 416 | Yes | Retrospective cohort |
| Wang, D. | Clinical analysis of 31 cases of 2019 novel coronavirus infection in children from six provinces (autonomous region) of northern China. [Chinese] | China | Multi-city | . | 25 January 2020 to 21 February 2020 | Pediatric patients with COVID19 | 31 | No | Retrospective cohort |
| Yang, X. | Clinical course and outcomes of critically ill patients with SARS-CoV-2 pneumonia in Wuhan, China: a single-centered, retrospective, observational study | China | Wuhan | Jin-Yin-tan Hospital | 24 December 2019 to 26 January 2020 | COVID19 patients admitted to the intensive care unit | 52 | Yes | Retrospective cohort |
| Zhu, L. | Coronavirus Disease 2019 Pneumonia in Immunosuppressed Renal Transplant Recipients: A Summary of 10 Confirmed Cases in Wuhan, China | China | Wuhan | Tongji Hospital | Until 16 March 2020 | COVID19 patients with kidney transplant | 10 | No | Retrospective cohort |
| Chen, T. | Clinical characteristics of 113 deceased patients with coronavirus disease 2019: retrospective study | China | Wuhan | Tongji Hospital | 13 January 2020 to 12 February 2020 | General population with COVID19 | 274 | Yes | Case-control |
| Zhang, W. | Clinical characteristics of 74 hospitalized patients with COVID-19 [Chinese] | China | Beijing | Beijing Youan Hospital | 21 January 2020 to 11 February 2020 | General population with COVID19 | 74 | No | Retrospective cohort |
| Wang, X. | Extrapulmonary organ damage and clinical significance in patients with coronavirus disease 2019 | China | Hangzhou | Hangzhou Xixi Hospital | 23 January 2020 to 24 February 2020 | General population with COVID19 | 72 | No | Retrospective cohort |
| Argenziano, MG | Characterization and Clinical Course of 1000 Patients with COVID-19 in New York: retrospective case series | USA | New York City | New York-Presbyterian / Columbia University Irving Medical Center | 1 March 2020 to 15 April 2020 | General population with COVID19 | 1000 | Yes | Retrospective cohort |
| Zhou, F | Clinical course and risk factors for mortality of adult inpatients with COVID-19 in Wuhan, China: a retrospective cohort study | China | Wuhan | Jinyintan Hospital, Wuhan Pulmonary Hospital | 29 December 2019 to 31 January 2020 | General population with COVID19 | 191 | Yes | Retrospective cohort |
| Kular, et al. | The characteristics, dynamics and the risk of death in COVID-19 positive dialysis patients in London, UK | UK | London | 3 South London NHS renal centres | 29 February 2020 to 15 May 2020 | COVID19 Patients with dialysis | 224 | Yes | Retrospective cohort |
| The Columbia University Kidney Transplant Program | Early Description of Coronavirus 2019 Disease in Kidney Transplant Recipients in New York | USA | New York | Columbia University Medical Center | Up to 27 March 2020 | COVID19 patients with kidney transplant | 15 | Yes | Retrospective cohort |
| Akalin, Enver | Covid-19 and Kidney Transplantation | USA | New York | Montefiore | 16 March 2020 to 1 April 2020 | COVID 19patients with kidney transplant | 36 | Yes | Retrospective cohort |
| Alberici, Federico | A single center observational study of the clinical characteristics and short-term outcome of 20 kidney transplant patients admitted for SARS-CoV2 pneumonia | Italy | Brescia | Spedali Civili Hospital | 27 February 2020 to 24 March 2020 | COVID19 patients with kidney transplant | 20 | Yes | Retrospective cohort |
| Pei, G | Renal Involvement and Early Prognosis in Patients with COVID-19 Pneumonia | China | Wuhan | Tongji Hospital | 28 January 2020 to 9 February 2020 | General population with COVID19. Pediatric patients and patients with a history of maintenance dialysis or renal transplantation were excluded | 333 | No | Retrospective cohort |
| Trujillo, H | SARS-CoV-2 Infection in Hospitalized Patients with Kidney Disease | Spain | Madrid | University Hospital "12 de Octubre" | . | COVID 19 patients with dialysis or transplant history | 51 | No | Retrospective cohort |
| Zhao, X | Clinical characteristics of patients with 2019 coronavirus disease in a non-Wuhan area of Hubei Province, China: a retrospective study | China | Wuhan | Jingzhou Central Hospital | 16 January 2020 to 10 February 2020 | General population with COVID19 | 91 | No | Retrospective cohort |
| Arentz, M | Characteristics and Outcomes of 21 Critically Ill Patients With COVID-19 in Washington State | USA | King and Snohomish Counties | Evergreen Hospital | 20 February 2020 to 5 March 2020 | COVID19 patients admitted to ICU | 21 | No | Retrospective cohort |
| Richardson, S | Presenting Characteristics, Comorbidities, and Outcomes Among 5700 Patients Hospitalized With COVID-19 in the New York City Area | USA | New York City, Long Island, Westchester County | North Shore University Hospital, Long Island Jewish Medical Center, Staten Island University Hospital, Lenox Hill Hospital, Southside Hospital, Huntington Hospital, Long Island Jewish Forest Hills, Long Island Jewish Valley Stream, Plainview Hospital, Cohen Children's Medical Center, Glen Cove Hospital, Syosset Hospital | 1 March 2020 to 4 April 2020 | General population with COVID19 | 2634 | Yes | Retrospective cohort |
| Grein, J | Compassionate Use of Remdesivir for Patients with Severe Covid-19 | Germany | Multi-city | Mackenzie Health (Canada); University Hospital of Bordeaux (France); Universitätsklinikum Düsseldorf (Germany); Università di Pavia, Spedali Civili Hospital, National Institute for Infectious Diseases, San Gerardo Hospital, Fondazione IRCCS Policlinico San Matteo, San Raffaele Institute, Azienda USL-IRCCS Reggio Emilia, (Italy); Tokyo Bay Urayasu Ichikawa Medical Center, Tosei General Hospital, Gunma University Hospital, Hiratsuka City Hospital, National Center for Global Health and Medicine (Japan); Hospital Universitario La Paz- Carlos III (Spain); San Bernardino Regional Medical Cente; United States Public Health Service Commissioned Corps, Miriam Hospital, El Camino Hospital, Providence Regional Medical Center Everett, Virginia Mason Medical Center, University of Washington Medical Center, UC Davis Health, John Muir Health (US); Kaiser Franz Josef Hospital (Austria) | 25 January 2020 to 7 March 2020 | Severe COVID19 patients (oxygen saturation of 94% or less while the patient was breathing ambient air or a need for oxygen support) on remdesivir | 53 | Yes | Retrospective cohort |
| Guan, W | Clinical Characteristics of Coronavirus Disease 2019 in China | China | Multi-city | 552 hospitals | 11 December 2019 to 31 January 2020 | General population with COVID19 | 1099 | No | Retrospective cohort |
| Hirsch, JS. | Acute kidney injury in patients hospitalized with COVID-19 | USA | New York City | 13 Northwell Hospitals | 1 March 2020 to 5 April 2020 | Adult general population with COVID19 | 5449 | Yes | Retrospective cohort |
| Shi, Q. | Clinical Characteristics and Risk Factors for Mortality of COVID-19 Patients With Diabetes in Wuhan, China: a Two-Center, Retrospective Study | China | Wuhan | Renmin Hospital, Zhongnan Hospital | 1 January 2020 to 8 March 2020 | Diabetes population | 153 | No | Retrospective cohort |
| Perez-Saez, M. | Use of tocilizumab in kidney transplant recipients with COVID-19 | Spain | Multi-centre | 29 hospitals | 18 March 2020 to 9 May 2020 | Kidney transplant patients | 80 | No | Retrospective cohort |
| Lecronier, M. | Comparison of hydroxychloroquine, lopinavir/ritonavir, and standard of care in critically ill patients with SARS-CoV-2 pneumonia: an opportunistic retrospective analysis | France | Paris | Hopital Pitie-Salpetriere | 4 March 2020 to 6 April 2020 | COVID19 patients admitted to ICU | 80 | No | Retrospective cohort |
| Alattar, R. | Tocilizumab for the treatment of severe coronavirus disease 2019 | Qatar | . | . | . | ICU COVID19 treated with tocilizumab | 25 | No | Retrospective cohort |
| Portolés, J. | Chronic kidney disease and acute kidney injury in the COVID-19 Spanish outbreak | Spain | Madrid | Puerta de Hierro Hospital | 25 February 2020 to 24 April 2020 | General population with COVID19 | 1603 | Yes | Prospective cohort |
| Palmieri, L. | Clinical Characteristics of Hospitalized Individuals Dying With COVID-19 by Age Group in Italy | Italy | Multi-centre | Multiple hospitals | 20 February 2020 to 21 May 2020 | Deceased COVID19 patients | 3032 | No | Retrospective cohort |
| Lendorf, M. | Characteristics and early outcomes of patients hospitalised for COVID-19 in North Zealand, Denmark | Denmark | . | North Zealand Hospital | 1 March 2020 to 4 May 2020 | General population with COVID19 | 111 | Yes | Retrospective cohort |
| Lam, K. | Continued In-Hospital Angiotensin-Converting Enzyme Inhibitor and Angiotensin II Receptor Blocker Use in Hypertensive COVID-19 Patients Is Associated With Positive Clinical Outcome | USA | New York | Stony Brook Hospital | 7 February 2020 to 23 May 2020 | Hypertensive COVID-19 patients | 614 | No | Retrospective cohort |
| Karagiannidis, C. | Case characteristics, resource use, and outcomes of 10 021 patients with COVID-19 admitted to 920 German hospitals: an observational study | Germany | Multi-centre | 920 hospitals | 26 February 2020 to 19 April 2020 | General population with COVID19 | 10021 | N/A | Retrospective cohort |
| Imam, Z. | Older age and comorbidity are independent mortality predictors in a large cohort of 1305 COVID-19 patients in Michigan, United States | USA | Detroit | Beaumont Health's 8 hospitals | 1 March 2020 to 1 April 2020 | General population with COVID19 | 1305 | Yes | Retrospective cohort |
| Azoulay, E. | Increased mortality in patients with severe SARS-CoV-2 infection admitted within seven days of disease onset | France | Paris | Hopital Saint-Louis, Hopital Tenon, Hopital Ambroise, Hopital Pitie-Salpetriere | 21 February 2020 to 24 April 2020 | COVID19 patients admitted to ICU | 379 | No | Retrospective cohort |
| Zhang, L. | Analysis of the clinical characteristics of patients infected with novel coronavirus pneumonia in China | China | Chengdu | Public Health Clinical Center of Chengdu | 16 January 2020 to 16 February 2020 | General population with COVID19 | 101 | No | Retrospective cohort |
| Liu, Y. | Clinical analysis of kidney injury in patients with COVID-19 | China | Wuhan | Central Theater Command General Hospital of Chinese PLA | 12 March 2020 | General population with COVID19 | 87 | No | Cross-sectional |
| Lin, L. | Clinical analysis of novel coronavirus pneumonia complicated with acute kidney injury [Chinese] | China | Guangzhou | Guangzhou 8th People's Hospital | 20 January 2020 to 16 February 2020 | General population with COVID19 | 243 | N/A | Retrospective cohort |
| Lu, J. | Clinical feature analysis on death cases of the COVID-19 | China | Wuhan | Wuhan Fourth Hospital | January 2020 to March 2020 | Deceased COVID19 patients | 73 | No | Retrospective cohort |
| Li, A. | Analysis of clinical characteristics and prognostic factors in COVID-19 patients with cardiovascular disease | China | Beijing | Youan Hospital | 21 January 2020 to 24 February 2020 | COVID-19 patients with cardiovascular disease | 99 | No | Retrospective cohort |
| Zhao, M. | Comparison of clinical characteristics and outcomes of patients with coronavirus disease 2019 at different ages | China | Wuhan | Shouyi and East districts of Renmin Hospital | 1 January 2020 to 14 February 2020 | General population with COVID19 | 1000 | Yes | Retrospective cohort |
| Song, J. | A Comparison of Clinical Characteristics and Outcomes in Elderly and Younger Patients with COVID-19 | China | Wuhan | Puai Hospital | 14 January 2020 to 26 February 2020 | General population with COVID19 | 69 | No | Retrospective cohort |
| Na, K. | Acute Kidney Injury and Kidney Damage in COVID-19 Patients | Korea | Daejeon | Chungnam National University Hospital | 21 February 2020 to 24 April 2020 | General population with COVID19 | 66 | No | Retrospective cohort |
| Lubetzky, M. | Kidney allograft recipients, immunosuppression, and coronavirus disease-2019: a report of consecutive cases from a New York City transplant center | USA | New York | New York Presbyterian Hospital | 13 March 2020 to 20 April 2020 | COVID-19 patients with kidney transplant | 54 | No | Retrospective cohort |
| Husain-Syed, F. | Acute kidney injury and urinary biomarkers in hospitalized patients with coronavirus disease-2019 | Germany | Giessen | University Hospital of Giessen and Marburg | Since 21 April 2020 | General population with COVID19 | 23 | No | Retrospective cohort |
| He, F. | Risk factors for severe cases of COVID-19: a retrospective cohort study | China | Guangzhou | Guangzhou 8th People's Hospital | 15 January 2020 to 10 March 2020 | General population with COVID19 | 288 | Yes | Retrospective cohort |
| Gao, S. | Risk factors influencing the prognosis of elderly patients infected with COVID-19: a clinical retrospective study in Wuhan, China | China | Wuhan | Wuhan Third Hospital | 23 January 2020 to 29 February 2020 | General population with COVID19 | 210 | Yes | Retrospective cohort |
| Aggarwal, A. | Clinical and Epidemiological Features of SARS-CoV-2 Patients in SARI Ward of a Tertiary Care Centre in New Delhi | India | New Delhi | Dr Ram Manohar Lohia Hospital | 10 April 2020 to 30 April 2020 | General population with COVID19 | 32 | Yes | Retrospective cohort |
| Aggarwal, S. | Clinical features, laboratory characteristics, and outcomes of patients hospitalized with coronavirus disease 2019 (COVID-19): Early report from the United States | USA | Des Moines | Hospital system | Until 4 April 2020 | General population with COVID19 | 16 | Yes | Retrospective cohort |
| Alamdari, N. | Mortality Risk Factors among Hospitalized COVID-19 Patients in a Major Referral Center in Iran | Iran | Tehran | Shahid Modarres Hospital | 30 January to 5 April 2020 | General population with COVID19 | 459 | Yes | Retrospective cohort |
| Antinori, S. | Compassionate remdesivir treatment of severe Covid-19 pneumonia in intensive care unit (ICU) and Non-ICU patients: Clinical outcome and differences in post-treatment hospitalisation status | Italy | Milan | ASST Fatebenefratelli-Sacco | 23 February to 20 March 2020 | General population with COVID19 | 35 | No | Prospective cohort |
| Chand, S. | COVID-19-Associated Critical Illness-Report of the First 300 Patients Admitted to Intensive Care Units at a New York City Medical Center | USA | New York | Montefiore | 10 March 2020 to 11 April 2020 | COVID19 patients admitted to ICU | 300 | Yes | Retrospective cohort |
| Chen, Y. | [Predictive value of neutrophil/lymphocyte ratio on myocardial injury in severe COVID-19 patients] | China | Wuhan | Renmin Hospital | 30 January 2020 to 18 February 2020 | General population with COVID19 | 133 | Yes | Retrospective cohort |
| Deng, Y. | Clinical characteristics of fatal and recovered cases of coronavirus disease 2019 in Wuhan, China: a retrospective study | China | Wuhan | Hankou and Caidian branch, Tongji Hospital | 1 January 2020 to 21 February 2020 | General population with COVID19 | 225 | No | Case-control |
| Fisher, M. | AKI in Hospitalized Patients with and without COVID-19: A Comparison Study | USA | New York | Montefiore Health System | 11 March 2020 to 26 April 2020 | General population with COVID19 | 3345 | Yes | Retrospective cohort |
| Hong, K. | Clinical Features and Outcomes of 98 Patients Hospitalized with SARS-CoV-2 Infection in Daegu, South Korea: A Brief Descriptive Study | Korea | Daegu | Yeungnam University | Up to 29 March 2020 | General population with COVID19 | 98 | No | Retrospective cohort |
| Lu, J. | Clinical characteristics and outcomes of adult critically ill patients with COVID-19 in Honghu, Hubei Province | China | Jingzhou | Honghu People's Hospital of Jingzhou City | 19 January 2020 to 8 March 2020 | COVID19 patients admitted to ICU | 20 | No | Retrospective cohort |
| Melgosa, M. | SARS-CoV-2 infection in Spanish children with chronic kidney pathologies | Spain | Multi-centre | Multiple hospitals | 1 March 2020 to 15 April 2020 | COVID19 patients under 18 with chronic renal pathology | 16 | Yes | Retrospective cohort |
| Nair, V. | COVID-19 in kidney transplant recipients | USA | New York | 12 acute care hospitals in Northwell Health | 1 March 2020 to 27 March 2020 | COVID19 patients with kidney transplant | 10 | N/A | Prospective cohort |
| Nakeshbandi, M. | The impact of obesity on COVID-19 complications: a retrospective cohort study | USA | New York | State University of New York Downstate Health Sciences University | 10 March 2020 to 13 April 2020 | General population with COVID19 | 504 | Yes | Retrospective cohort |
| Oualha, M. | Severe and fatal forms of COVID-19 in children | France | Paris | Necker-Enfants-Malades University Hospital | 10 February 2020 to 20 April 2020 | Children with COVID19 | 27 | Yes | Retrospective cohort |
| Palaiodimos, L. | Severe obesity, increasing age and male sex are independently associated with worse in-hospital outcomes, and higher in-hospital mortality, in a cohort of patients with COVID-19 in the Bronx, New York | USA | New York | Montefiore | 9 March 2020 to 22 March 2020 | General population with COVID19 | 200 | Yes | Retrospective cohort |
| Pelayo, J. | Clinical Characteristics and Outcomes of Community- and Hospital-Acquired Acute Kidney Injury with COVID-19 in a US Inner City Hospital System | USA | . | A tertiary inner-city hospital | . | General population with COVID19 | 223 | Yes | Retrospective cohort |
| Pan, W. | Clinical Features of COVID-19 in Patients With Essential Hypertension and the Impacts of Renin-angiotensin-aldosterone System Inhibitors on the Prognosis of COVID-19 Patients | China | Wuhan | Renmin Hospital | 4 January 2020 to 14 February 2020 | General population with COVID19 | 996 | Yes | Retrospective cohort |
| Price-Haywood, E. | Hospitalization and Mortality among Black Patients and White Patients with Covid-19 | USA | New Orleans | Ochsner Health facility | 1 March 2020 to 11 April 2020 | General population with COVID19 | 1382 | Yes | Retrospective cohort |
| Soh, T. | Clinical characteristics of severe acute respiratory syndrome Coronavirus 2 (SARS-CoV2) patients in Hospital Tengku Ampuan Afzan | Malaysia | Kuantan | Hospital Tengku Ampuan Afzan | 9 March 2020 to 15 April 2020 | General population with COVID19 | 247 | N/A | . |
| Suleyman, G. | Clinical Characteristics and Morbidity Associated With Coronavirus Disease 2019 in a Series of Patients in Metropolitan Detroit | USA | Metropolitan Detroit | Henry Ford Health System | 6 March 2020 to 27 March 2020 | General population with COVID19 | 355 | Yes | Retrospective cohort |
| Sun, D. | Subclinical Acute Kidney Injury in COVID-19 Patients: A Retrospective Cohort Study | China | Wuxi | The Affiliated Wuxi No. 2 People's Hospital of Nanjing Medical University | . | General population with COVID19 | 32 | Yes | Retrospective cohort |
| Trabulus, S. | Kidney function on admission predicts in-hospital mortality in COVID-19 | Turkey | Istanbul | A tertiary care university hospital | 15 March 2020 to 1 May 2020 | General population with COVID19 | 336 | Yes | Retrospective cohort |
| Valeri, A. | Presentation and Outcomes of Patients with ESKD and COVID-19 | USA | New York | Columbia University Irving Medical Center | 9 March 2020 to 8 April 2020 | COVID19 patients with end-stage kidney disease under dialysis | 59 | N/A | Retrospective cohort |
| Xia, P. | Clinicopathological Features and Outcomes of Acute Kidney Injury in Critically Ill COVID-19 with Prolonged Disease Course: A Retrospective Cohort | China | Wuhan | Sino-French New City Capus of Tongji Hospital | 5 February 2020 to 20 March 2020 | COVID19 patients admitted to ICU | 81 | Yes | Retrospective cohort |
| Xu, J. | Clinical course and predictors of 60-day mortality in 239 critically ill patients with COVID-19: a multicenter retrospective study from Wuhan, China | China | Wuhan | Wuhan Union Hospital, Jinyintan Hospital, Wuhan Third Hospital | 12 January 2020 to 3 February 2020 | COVID19 patients admitted to ICU | 239 | Yes | Retrospective cohort |
| Zheng, Y. | Clinical characteristics of 34 COVID-19 patients admitted to intensive care unit in Hangzhou, China | China | Zhejiang | The First Affiliated Hospital, Zhejiang University | 22 January 2020 to 5 March 2020 | COVID19 patients admitted to ICU | 34 | No | Retrospective cohort |
| Ziehr, D. | Respiratory Pathophysiology of Mechanically Ventilated Patients with COVID-19: A Cohort Study | USA | Boston | Massachusetts General Hospital and Beth Israel Deaconess Medical Center | 11 March 2020 to 30 March 2020 | COVID19 patients with respiratory failure on invasive mechanical ventilation | 66 | Yes | Retrospective cohort |
| Chan, K. | Mass screening is associated with low rates of acute kidney injury among COVID-19 patients in Hong Kong | China | Hong Kong SAR | Queen Mary Hospital, Princess Margaret Hospital, Queen Elizabeth Hospital, United Christian Hospital and Pamela Youde Nethersole Eastern Hospital | 15 January 2020 to 31 May 2020 | Adult general population with COVID19 | 591 | Yes | Retrospective cohort |

^*^ Severe cases were defined as (i) respiratory rate >30 breaths/min, (ii) oxygen saturation =< 93%, or (iii) PaO2/FiO2, ratio =< 300 mm Hg. Critical cases were defined as including >= 1 of the following criteria: shock; respiratory failure requiring mechanical ventilation; combination with other organ failures; and admission to intensive care unit.

^&^ Wuhan Tongji Hospital, Wuhan Pulmonary Hospital, Huangshi Central Hospital, Chongqing Southwest Hospital.

^#^ North Shore University Hospital, Long Island Jewish Medical Center, Staten Island University Hospital, Lenox Hill Hospital, Southside Hospital, Huntington Hospital, Long Island Jewish Forest Hills, Long Island Jewish Valley Stream, Plainview Hospital, Cohen Children's Medical Center, Glen Cove Hospital, Syosset Hospital.

^^^ Mackenzie Health (Canada); University Hospital of Bordeaux (France); Universitätsklinikum Düsseldorf (Germany); Università di Pavia, Spedali Civili Hospital, National Institute for Infectious Diseases, San Gerardo Hospital, Fondazione IRCCS Policlinico San Matteo, San Raffaele Institute, Azienda USL-IRCCS Reggio Emilia, (Italy); Tokyo Bay Urayasu Ichikawa Medical Center, Tosei General Hospital, Gunma University Hospital, Hiratsuka City Hospital, National Center for Global Health and Medicine (Japan); Hospital Universitario La Paz- Carlos III (Spain); San Bernardino Regional Medical Cente; United States Public Health Service Commissioned Corps, Miriam Hospital, El Camino Hospital, Providence Regional Medical Center Everett, Virginia Mason Medical Center, University of Washington Medical Center, UC Davis Health, John Muir Health (US); Kaiser Franz Josef Hospital (Austria)

**S4 Quality assessment of included studies of meta-analysis**

|  |  | Selection | | | | Exposure and outcome | | | | |  |
| --- | --- | --- | --- | --- | --- | --- | --- | --- | --- | --- | --- |
| First Author | Title | Clear objective | Clear sampling definition | Consecutive / random sampling | Comparable controls (case-control / cohort) ^ | Clear COVID diagnosis | Clear outcome measures (AKI or RRT) | Adequate length of follow-up ^ | Clear statistical method | Clear description of result | Total |
| Cheng, Y. | Kidney disease is associated with in-hospital death of patients with COVID-19 | 1 | 1 | 1 | N/A | 1 | 1 | 1 | 1 | 1 | 8 |
| Ling, L. | Critically ill patients with COVID-19 in Hong Kong: a multicentre retrospective observational cohort study | 1 | 1 | 1 | N/A | 1 | 1 | 1 | 1 | 1 | 8 |
| Shi, S. | Association of Cardiac Injury with Mortality in Hospitalized Patients with COVID-19 in Wuhan, China | 1 | 1 | 1 | 1 | 1 | 1 | 1 | 1 | 1 | 9 |
| Wang, D. | Clinical analysis of 31 cases of 2019 novel coronavirus infection in children from six provinces (autonomous region) of northern China. [Chinese] | 1 | 1 | 0 | N/A | 1 | 1 | 1 | 1 | 1 | 7 |
| Yang, X. | Clinical course and outcomes of critically ill patients with SARS-CoV-2 pneumonia in Wuhan, China: a single-centered, retrospective, observational study | 1 | 1 | 1 | 1 | 1 | 1 | 1 | 1 | 1 | 9 |
| Zhu, L. | Coronavirus Disease 2019 Pneumonia in Immunosuppressed Renal Transplant Recipients: A Summary of 10 Confirmed Cases in Wuhan, China | 1 | 1 | 0 | 1 | 1 | 1 | 1 | 1 | 1 | 8 |
| Chen, T. | Clinical characteristics of 113 deceased patients with coronavirus disease 2019: retrospective study | 1 | 0 | 1 | 1 | 1 | 1 | 1 | 1 | 1 | 8 |
| Zhang, W. | Clinical characteristics of 74 hospitalized patients with COVID-19 [Chinese] | 1 | 1 | 0 | N/A | 1 | 1 | CD | 1 | 1 | 6 |
| Wang, X. | Extrapulmonary organ damage and clinical significance in patients with coronavirus disease 2019 | 1 | 1 | 0 | N/A | 1 | 1 | 1 | 1 | 1 | 7 |
| Argenziano, MG | Characterization and Clinical Course of 1000 Patients with COVID-19 in New York: retrospective case series | 1 | 1 | 1 | N/A | 1 | 1 | 1 | 0 | 1 | 7 |
| Zhou, F | Clinical course and risk factors for mortality of adult inpatients with COVID-19 in Wuhan, China: a retrospective cohort study | 1 | 1 | 1 | 1 | 1 | 1 | 1 | 1 | 1 | 9 |
| Kular, et al. | The characteristics, dynamics and the risk of death in COVID-19 positive dialysis patients in London, UK | 1 | 1 | 1 | N/A | 1 | 1 | CD | 1 | 1 | 7 |
| The Columbia University Kidney Transplant Program | Early Description of Coronavirus 2019 Disease in Kidney Transplant Recipients in New York | 1 | 1 | 1 | N/A | 0 | 1 | 0 | 0 | 1 | 5 |
| Akalin, Enver | Covid-19 and Kidney Transplantation | 1 | 1 | 1 | N/A | 0 | 1 | CD | 0 | 1 | 5 |
| Alberici, Federico | A single center observational study of the clinical characteristics and short-term outcome of 20 kidney transplant patients admitted for SARS-CoV2 pneumonia | 1 | 1 | 1 | N/A | 0 | 1 | 1 | 0 | 1 | 6 |
| Pei, G | Renal Involvement and Early Prognosis in Patients with COVID-19 Pneumonia | 1 | 1 | 0 | N/A | 1 | 1 | 1 | 1 | 1 | 7 |
| Trujillo, H | SARS-CoV-2 Infection in Hospitalized Patients with Kidney Disease | 1 | 0 | 0 | 1 | 1 | 1 | 1 | 0 | 1 | 6 |
| Zhao, X | Clinical characteristics of patients with 2019 coronavirus disease in a non-Wuhan area of Hubei Province, China: a retrospective study | 1 | 1 | 0 | N/A | 1 | 1 | CD | 1 | 1 | 6 |
| Arentz, M | Characteristics and Outcomes of 21 Critically Ill Patients With COVID-19 in Washington State | 1 | 1 | 0 | N/A | 1 | 1 | 0 | 1 | 1 | 6 |
| Richardson, S | Presenting Characteristics, Comorbidities, and Outcomes Among 5700 Patients Hospitalized With COVID-19 in the New York City Area | 1 | 1 | 1 | N/A | 1 | 1 | 0 | 1 | 1 | 7 |
| Grein, J | Compassionate Use of Remdesivir for Patients with Severe Covid-19 | 1 | 1 | 1 | N/A | 1 | 1 | 1 | 1 | 1 | 8 |
| Guan, W | Clinical Characteristics of Coronavirus Disease 2019 in China | 1 | 1 | 0 | N/A | 1 | 1 | 1 | 1 | 1 | 7 |
| Lin, L | Clinical analysis of novel coronavirus pneumonia complicated with acute kidney injury [Chinese] | 1 | 1 | 0 | N/A | 1 | 1 | 1 | 1 | 1 | 7 |
| Hirsch, JS. | Acute kidney injury in patients hospitalized with COVID-19 | 1 | 0 | 1 | 1 | 0 | 1 | 1 | 1 | 1 | 7 |
| Shi, Q. | Clinical Characteristics and Risk Factors for Mortality of COVID-19 Patients With Diabetes in Wuhan, China: a Two-Center, Retrospective Study | 1 | 1 | 0 | N/A | 1 | 1 | CD | 1 | 1 | 6 |
| Perez-Saez, M. | Use of tocilizumab in kidney transplant recipients with COVID-19 | 1 | 1 | 0 | N/A | 1 | 0 | 1 | 1 | 1 | 6 |
| Lecronier, M. | Comparison of hydroxychloroquine, lopinavir/ritonavir, and standard of care in critically ill patients with SARS-CoV-2 pneumonia: an opportunistic retrospective analysis | 1 | 1 | 0 | N/A | 1 | 1 | 1 | 1 | 1 | 7 |
| Alattar, R. | Tocilizumab for the treatment of severe coronavirus disease 2019 | 1 | 1 | 0 | N/A | 1 | 1 | 1 | 1 | 1 | 7 |
| Portolés, J. | Chronic kidney disease and acute kidney injury in the COVID-19 Spanish outbreak | 1 | 1 | 1 | 1 | 1 | 1 | CD | 1 | 1 | 8 |
| Palmieri, L. | Clinical Characteristics of Hospitalized Individuals Dying With COVID-19 by Age Group in Italy | 1 | 1 | 0 | N/A | 1 | 0 | CD | 1 | 1 | 5 |
| Lendorf, M. | Characteristics and early outcomes of patients hospitalised for COVID-19 in North Zealand, Denmark | 1 | 1 | 1 | N/A | 1 | 1 | 1 | 1 | 1 | 8 |
| Lam, K. | Continued In-Hospital Angiotensin-Converting Enzyme Inhibitor and Angiotensin II Receptor Blocker Use in Hypertensive COVID-19 Patients Is Associated With Positive Clinical Outcome | 1 | 1 | 0 | N/A | 1 | 0 | CD | 1 | 1 | 5 |
| Karagiannidis, C. | Case characteristics, resource use, and outcomes of 10 021 patients with COVID-19 admitted to 920 German hospitals: an observational study | 1 | 1 | 0 | N/A | 1 | 1 | CD | 1 | 1 | 6 |
| Imam, Z. | Older age and comorbidity are independent mortality predictors in a large cohort of 1305 COVID-19 patients in Michigan, United States | 1 | 1 | 1 | N/A | 1 | 1 | CD | 1 | 1 | 7 |
| Azoulay, E. | Increased mortality in patients with severe SARS-CoV-2 infection admitted within seven days of disease onset | 1 | 1 | 0 | N/A | 1 | 1 | 1 | 1 | 1 | 7 |
| Zhang, L. | Analysis of the clinical characteristics of patients infected with novel coronavirus pneumonia in China | 1 | 1 | 0 | N/A | 1 | 0 | CD | 1 | 1 | 5 |
| Liu, Y. | Clinical analysis of kidney injury in patients with COVID-19 | 1 | 1 | 0 | N/A | 1 | 1 | 1 | 1 | 1 | 7 |
| Lu, J. | Clinical feature analysis on death cases of the COVID-19 | 1 | 1 | 0 | N/A | 1 | 0 | CD | 1 | 1 | 5 |
| Li, A. | Analysis of clinical characteristics and prognostic factors in COVID-19 patients with cardiovascular disease | 1 | 1 | 0 | N/A | 1 | 0 | CD | 1 | 1 | 5 |
| Zhao, M. | Comparison of clinical characteristics and outcomes of patients with coronavirus disease 2019 at different ages | 1 | 1 | 1 | N/A | 1 | 1 | CD | 1 | 1 | 7 |
| Song, J. | A Comparison of Clinical Characteristics and Outcomes in Elderly and Younger Patients with COVID-19 | 1 | 1 | 0 | N/A | 1 | 0 | 1 | 1 | 1 | 6 |
| Na, K. | Acute Kidney Injury and Kidney Damage in COVID-19 Patients | 1 | 1 | 0 | N/A | 1 | 1 | CD | 1 | 1 | 6 |
| Lubetzky, M. | Kidney allograft recipients, immunosuppression, and coronavirus disease-2019: a report of consecutive cases from a New York City transplant center | 1 | 1 | 0 | N/A | 1 | 1 | 1 | 1 | 1 | 7 |
| Husain-Syed, F. | Acute kidney injury and urinary biomarkers in hospitalized patients with coronavirus disease-2019 | 1 | 1 | 0 | N/A | 1 | 1 | CD | 1 | 1 | 6 |
| He, F. | Risk factors for severe cases of COVID-19: a retrospective cohort study | 1 | 1 | 1 | N/A | 1 | 1 | CD | 1 | 1 | 7 |
| Gao, S. | Risk factors influencing the prognosis of elderly patients infected with COVID-19: a clinical retrospective study in Wuhan, China | 1 | 1 | 1 | N/A | 1 | 1 | 1 | 1 | 1 | 8 |
| Aggarwal, A. | Clinical and Epidemiological Features of SARS-CoV-2 Patients in SARI Ward of a Tertiary Care Centre in New Delhi | 1 | 1 | 1 | N/A | 1 | 1 | 1 | 1 | 1 | 8 |
| Aggarwal, S. | Clinical features, laboratory characteristics, and outcomes of patients hospitalized with coronavirus disease 2019 (COVID-19): Early report from the United States | 1 | 1 | 1 | N/A | 1 | 0 | CD | 1 | 1 | 6 |
| Alamdari, N. | Mortality Risk Factors among Hospitalized COVID-19 Patients in a Major Referral Center in Iran | 1 | 1 | 1 | N/A | 1 | 0 | CD | 1 | 1 | 6 |
| Antinori, S. | Compassionate remdesivir treatment of severe Covid-19 pneumonia in intensive care unit (ICU) and Non-ICU patients: Clinical outcome and differences in post-treatment hospitalisation status | 1 | 1 | 0 | N/A | 1 | 0 | 1 | 1 | 1 | 6 |
| Chand, S. | COVID-19-Associated Critical Illness-Report of the First 300 Patients Admitted to Intensive Care Units at a New York City Medical Center | 1 | 1 | 1 | N/A | 1 | 1 | 1 | 1 | 1 | 8 |
| Chen, Y. | [Predictive value of neutrophil/lymphocyte ratio on myocardial injury in severe COVID-19 patients] | 1 | 1 | 1 | N/A | 1 | 1 | CD | 1 | 1 | 7 |
| Deng, Y. | Clinical characteristics of fatal and recovered cases of coronavirus disease 2019 in Wuhan, China: a retrospective study | 1 | 1 | 0 | N/A | 1 | 0 | 1 | 1 | 1 | 6 |
| Fisher, M. | AKI in Hospitalized Patients with and without COVID-19: A Comparison Study | 1 | 1 | 1 | N/A | 1 | 1 | CD | 1 | 1 | 7 |
| Hong, K. | Clinical Features and Outcomes of 98 Patients Hospitalized with SARS-CoV-2 Infection in Daegu, South Korea: A Brief Descriptive Study | 1 | 1 | 0 | N/A | 1 | 1 | CD | 1 | 1 | 6 |
| Lu, J. | Clinical characteristics and outcomes of adult critically ill patients with COVID-19 in Honghu, Hubei Province | 1 | 1 | 0 | N/A | 1 | 0 | CD | 1 | 1 | 5 |
| Melgosa, M. | SARS-CoV-2 infection in Spanish children with chronic kidney pathologies | 1 | 1 | 1 | N/A | 1 | 0 | 1 | 1 | 1 | 7 |
| Nair, V. | COVID-19 in kidney transplant recipients | 1 | 1 | 0 | N/A | 1 | 1 | 1 | 1 | 1 | 7 |
| Nakeshbandi, M. | The impact of obesity on COVID-19 complications: a retrospective cohort study | 1 | 1 | 1 | N/A | 1 | 1 | CD | 1 | 1 | 7 |
| Oualha, M. | Severe and fatal forms of COVID-19 in children | 1 | 1 | 1 | N/A | 1 | 1 | CD | 1 | 1 | 7 |
| Palaiodimos, L. | Severe obesity, increasing age and male sex are independently associated with worse in-hospital outcomes, and higher in-hospital mortality, in a cohort of patients with COVID-19 in the Bronx, New York | 1 | 1 | 1 | N/A | 1 | 0 | 1 | 1 | 1 | 7 |
| Pelayo, J. | Clinical Characteristics and Outcomes of Community- and Hospital-Acquired Acute Kidney Injury with COVID-19 in a US Inner City Hospital System | 1 | 1 | 1 | N/A | 1 | 1 | CD | 1 | 1 | 7 |
| Pan, W. | Clinical Features of COVID-19 in Patients With Essential Hypertension and the Impacts of Renin-angiotensin-aldosterone System Inhibitors on the Prognosis of COVID-19 Patients | 1 | 1 | 1 | N/A | 1 | 1 | CD | 1 | 1 | 7 |
| Price-Haywood, E. | Hospitalization and Mortality among Black Patients and White Patients with Covid-19 | 1 | 1 | 1 | N/A | 1 | 0 | 1 | 1 | 1 | 7 |
| Soh, T. | Clinical characteristics of severe acute respiratory syndrome Coronavirus 2 (SARS-CoV2) patients in Hospital Tengku Ampuan Afzan | 1 | 1 | 0 | N/A | 1 | 1 | 1 | 1 | 1 | 7 |
| Suleyman, G. | Clinical Characteristics and Morbidity Associated With Coronavirus Disease 2019 in a Series of Patients in Metropolitan Detroit | 1 | 1 | 1 | N/A | 1 | 1 | 1 | 1 | 1 | 8 |
| Sun, D. | Subclinical Acute Kidney Injury in COVID-19 Patients: A Retrospective Cohort Study | 1 | 1 | 1 | N/A | 1 | 1 | CD | 1 | 1 | 7 |
| Trabulus, S. | Kidney function on admission predicts in-hospital mortality in COVID-19 | 1 | 1 | 1 | N/A | 1 | 1 | CD | 1 | 1 | 7 |
| Valeri, A. | Presentation and Outcomes of Patients with ESKD and COVID-19 | 1 | 1 | 0 | N/A | 1 | 1 | 1 | 1 | 1 | 7 |
| Xia, P. | Clinicopathological Features and Outcomes of Acute Kidney Injury in Critically Ill COVID-19 with Prolonged Disease Course: A Retrospective Cohort | 1 | 1 | 1 | N/A | 1 | 1 | CD | 1 | 1 | 7 |
| Xu, J. | Clinical course and predictors of 60-day mortality in 239 critically ill patients with COVID-19: a multicenter retrospective study from Wuhan, China | 1 | 1 | 1 | N/A | 1 | 1 | 1 | 1 | 1 | 8 |
| Zheng, Y. | Clinical characteristics of 34 COVID-19 patients admitted to intensive care unit in Hangzhou, China | 1 | 1 | 0 | N/A | 1 | 1 | CD | 1 | 1 | 6 |
| Ziehr, D. | Respiratory Pathophysiology of Mechanically Ventilated Patients with COVID-19: A Cohort Study | 1 | 1 | 1 | N/A | 1 | 1 | 1 | 1 | 1 | 8 |
| Chan, K. | Longitudinal REnal involvement of CORonavirus Disease 2019 | 1 | 1 | 1 | N/A | 1 | 1 | 1 | 1 | 1 | 8 |

^ NA: Not applicable. CD: Could not determine

**S5 Global incidence of renal manifestations therapy among COVID-19 patients with no history of renal replacement therapy**

The pooled incidence of renal replacement therapy (RRT) (17 studies, n=18,569) were 2.97%. The prevalence of proteinuria (5 studies, n=11,130) and hematuria (3 studies, n=7,753) were 52.09% and 45.38%, respectively.

**S6 Odds ratios of acute kidney injury with critical presentation among COVID-19 patients with no history of renal replacement therapy**

**S7 Odds ratios of renal replacement therapy with mortality and critical presentation among COVID-19 patients with no history of renal replacement therapy**

Start of RRT was associated with 18.7 times increased odds of mortality and 34.0 times increased odds of critical presentation.

**S8 Association between clinical presentations and odds ratio of acute kidney injury and mortality**

Studies with higher percentage of patients presented with dyspnoea (P<0.01), diarrhoea (P=0.01) and cough (P=0.04) were associated with a higher incidence of in-hospital acute kidney injury (AKI). Positive trends of AKI incidence with fatigue were observed but not fever.

**S9 Odds ratios of acute kidney injury with mortality among COVID-19 patients with history of renal replacement therapy**
